# Carbapenemases in Gram-Negative Bacilli: Classification, Detection, Therapeutic Options, and Resistance to Novel Antimicrobials

**DOI:** 10.3390/antibiotics15100986

**Published:** 2026-10-06

**Authors:** Silvana Vulpie, Petrinela Ceachir, Alexandru-Mihai Craciun, Ionica Varan, Oana Magiar, Dorina Dugăeșescu, Delia Muntean, Monica Licker

**Affiliations:** 1 Doctoral School, “Victor Babes” University of Medicine and Pharmacy, 300041 Timisoara, Romania; 2 Multidisciplinary Research Centre of Antimicrobial Resistance, Microbiology Department, “Victor Babes” University of Medicine and Pharmacy, 300041 Timisoara, Romania; 3 Microbiology Department, “Victor Babes” University of Medicine and Pharmacy, 300041 Timisoara, Romania; 4 Microbiology Laboratory, “Pius Branzeu” County Clinical Emergency Hospital, 300723 Timisoara, Romania; 5 Metagenomic Reference Center, “Victor Babes” University of Medicine and Pharmacy, 300041 Timisoara, Romania

**Keywords:** Gram-negative bacilli, carbapenem resistance, carbapenemases, β-lactam–β-lactamase inhibitor combinations, carbapenemase detection methods, treatment-emergent resistance

## Abstract

Carbapenem-resistant Gram-negative bacilli (CR-GNB), particularly Enterobacterales, *Pseudomonas aeruginosa*, and *Acinetobacter baumannii*, remain a major clinical challenge and serious threat to modern medicine, with carbapenemase production representing a particular clinical and epidemiological concern. This structured narrative review integrates three clinically linked areas: the evolution and classification of clinically important carbapenemases; phenotypic and molecular methods for their detection and characterization; and the organism- and enzyme-specific activity of recently introduced antimicrobial agents and β-lactam–β-lactamase inhibitor combinations, including ceftolozane–tazobactam, ceftazidime–avibactam, meropenem–vaborbactam, imipenem–cilastatin–relebactam, cefiderocol, plazomicin, eravacycline, cefepime–enmetazobactam, sulbactam–durlobactam (FDA 2023), aztreonam–avibactam (EMA 2024; FDA 2025), and cefepime–zidebactam (FDA 2026). Particular emphasis is placed on baseline, experimentally selected and treatment-emergent resistance mechanisms and their clinical implications. Although these agents have substantially expanded the therapeutic armamentarium against CR-GNB, important gaps persist, particularly for metallo-β-lactamase-producing organisms, carbapenem-resistant *A. baumannii*, and metallo-β-lactamase-producing *P. aeruginosa*. Their activity may be compromised by pre-existing resistance determinants, target alterations, permeability defects, efflux-pump overexpression, β-lactamase evolution or overproduction, and combinations of these mechanisms, while treatment-emergent resistance threatens long-term effectiveness. By linking laboratory carbapenemase detection with a comparative, mechanism-based assessment of antimicrobial activity, resistance determinants, and clinical limitations, this review provides a practical framework for mechanism-directed therapy and antimicrobial stewardship and identifies priorities for future research, including improved direct inhibition of metallo-β-lactamases, more reliable therapeutic options for CR-GNB, prospective studies stratified by resistance mechanisms, strengthened epidemiological surveillance and infection-prevention strategies, and continued therapeutic innovation.

## 1. Introduction

Gram-negative bacilli (GNB), including members of the order Enterobacterales and clinically relevant non-fermenting species such as *Pseudomonas aeruginosa* and *Acinetobacter baumannii*, are among the leading causes of both community-acquired and healthcare-associated infections (HAIs). The increasing prevalence of antimicrobial resistance (AMR) in these pathogens has become a major threat to modern medicine, substantially limiting therapeutic options and contributing to increased morbidity, mortality, healthcare costs, and prolonged hospital stay [1,2,3].

The emergence and dissemination of resistance among GNB are facilitated by their remarkable capacity to adapt under antimicrobial selective pressure. Resistance may develop either through chromosomal mutations or through the acquisition of resistance determinants by horizontal gene transfer, the latter playing a particularly important role in their dissemination between bacterial strains and species [2,4]. This process is mediated predominantly by plasmids and other mobile genetic elements (MGEs), including transposons, insertion sequences, and integrative and conjugative elements, frequently involving integrons and resistance gene cassettes [5].

Carbapenem-resistant Gram-negative bacilli (CR-GNB) represent a particularly serious clinical challenge because carbapenems have long served as key agents for treating severe infections caused by multidrug-resistant (MDR) GNB, particularly ESBL-producing Enterobacterales [6]. Their core structure consists of a β-lactam ring fused to an unsaturated five-membered ring containing a carbon atom in place of the sulfur present in penicillins. The β-lactam ring enables inhibition of penicillin-binding proteins (PBPs), disrupting peptidoglycan synthesis. The carboxyl group of the bicyclic core is required for recognition by these target enzymes. The hydroxyethyl substituent and the configuration of the carbapenem scaffold contribute to stability against many β-lactamases, while differences in side chains influence antibacterial and pharmacological properties [7]. The 1β-methyl group, present in meropenem, ertapenem, doripenem and tebipenem but absent in imipenem, confers stability against renal dehydropeptidase-I (DHP-I), which explains why imipenem is co-administered with cilastatin. The chemical structures of imipenem, meropenem, ertapenem, doripenem, and tebipenem are illustrated in Figure 1. Tebipenem pivoxil, the orally administered prodrug of tebipenem, received U.S. Food and Drug Administration (FDA) approval in June 2026, becoming the first oral carbapenem therapy approved in the United States [8].

Carbapenem resistance may be mediated by carbapenemase production or by species-dependent combinations of reduced outer-membrane permeability, efflux-pump overexpression, and production or overproduction of non-carbapenemase β-lactamases, particularly ESBLs and AmpC enzymes [9,10,11,12] (Figure 2). Among these mechanisms, carbapenemase production is of epidemiological concern because many acquired carbapenemase genes are carried on plasmids and/or embedded within other MGEs, facilitating their dissemination between bacterial strains and species [13,14,15]. Moreover, the same plasmids may also co-harbour determinants conferring resistance to other antimicrobial classes [16,17,18]. Consequently, carbapenemase-producing organisms frequently exhibit MDR, extensively drug-resistant (XDR) or even pandrug-resistant (PDR) phenotypes.

Recognising the growing public health impact of these resistant microorganisms, in 2017 the World Health Organization (WHO) classified carbapenem-resistant *A. baumannii* (CRAB), *P. aeruginosa* (CRPA), and Enterobacteriaceae (the taxonomic designation used in the 2017 list, subsequently reclassified as Enterobacterales) (CRE) as critical-priority pathogens for antibacterial research and development. Furthermore, in the revised 2024 list, CRAB and CRE remained within the critical-priority category, whereas CRPA was reclassified as a high-priority pathogen, as data showed some decrease in resistance in certain WHO regions. However, this pathogen remains of particular concern in other populations and regions; therefore, research and investments are still needed in order to develop new therapeutic options for the treatment of infections caused by CRPA [19,20,21].

Although the introduction of newer antimicrobial agents and β-lactam/β-lactamase inhibitor (BL/BLI) combinations has expanded the therapeutic options available for some carbapenem-resistant infections, important gaps in antimicrobial coverage remain. Their activity varies according to the bacterial species, carbapenemases produced, and coexisting resistance mechanisms. Moreover, the increasing recognition of both baseline and treatment-emergent resistance to these agents threatens their long-term clinical effectiveness and emphasizes the need for rapid and accurate diagnostic methods, antimicrobial stewardship, epidemiological surveillance, and continued therapeutic innovation.

Grossman and Thompson [7] recently reviewed carbapenemase molecular diversity and advances in BL/BLI therapy, including resistance mechanisms and gaps in coverage. The present structured narrative review complements their work by comparing phenotypic and molecular detection methods, their practical limitations, and their implications for treatment selection. It also broadens the therapeutic scope to include cefiderocol and non-β-lactam agents such as plazomicin and eravacycline, examining their activity, resistance mechanisms, and clinical limitations in the context of CR-GNB infections. Furthermore, we incorporated the two most recent regulatory events in this field—the approval of aztreonam–avibactam (EMA, April 2024; FDA, February 2025) [22,23] and cefepime–zidebactam (FDA, May 2026) [24], together with the in vitro evidence published for the latter against isolates already resistant to the earlier BL/BLI combinations.

## 2. Material and Method

This narrative review used PubMed as the bibliographic database. An initial search was conducted in July 2025 to identify the background literature published over the preceding 10 years. Additional topic-specific searches were performed during the writing of each section to incorporate updated evidence, focusing on publications from the preceding five years. The final search was completed in August 2026.

Searches addressed carbapenemase classification, laboratory detection, therapeutic options, and resistance to newer antimicrobial agents. The reference lists of representative articles were also screened to identify additional publications relevant to each section, including older studies providing historical or mechanistic context. Official FDA and European Medicines Agency (EMA) sources were consulted for antimicrobial approvals and indications, while WHO publications were consulted for antimicrobial-resistance priorities.

Only full-length articles published in English were eligible for inclusion. Publications were selected according to their relevance to the topics addressed in each section, and the evidence was synthesized narratively.

Generative AI tools were used in the preparation of the figures: BioRender AI (BioRender, Toronto, ON, Canada) was used to generate draft layouts of Figure 1 and Figure 2. The chemical structures in Figure 1 were drawn PubChem 2D coordinates (CIDs 104838, 441130, 150610, 73303 and 9800194), and all figures were checked and edited by the authors.

## 3. Carbapenemases

GNB may acquire resistance to carbapenems through mechanisms commonly associated with β-lactam resistance (Figure 2). These mainly include decreased expression or loss of outer membrane proteins, the porins (OMPs), particularly when combined with the overexpression of class C cephalosporinases (which are now frequently also plasmid-encoded, therefore encountered in species that are not known to have chromosomal AmpC genes), increased activity of efflux pumps and the production of carbapenemases [25]. Carbapenemases are β-lactamase enzymes that hydrolyze carbapenems, thereby inactivating these agents before they can reach their target sites, the penicillin-binding proteins (PBPs). In contrast, alterations in PBPs, another β-lactam resistance mechanism frequently seen in Gram-positive microorganisms, representing the main resistance mechanism towards β-lactams in these bacteria [26], are rarely implicated in carbapenem resistance among GNB. Instead, the production of drug-inactivating enzymes represents the predominant mechanism of carbapenem resistance in these organisms, consistent with resistance patterns observed for other β-lactam antibiotics [13]. Moreover, the genes encoding these enzymes are often located on plasmids and/or other MGEs, which facilitate horizontal gene transfer among GNB and substantially contribute to the emergence and dissemination of MDR pathogens, particularly within healthcare settings.

Initially, chromosomally-encoded carbapenemases, primarily MBLs, were identified mainly in bacteria isolated from environmental sources (e.g., Gram-positive *Bacillus* spp.) [27] or, sporadically, in *Stenotrophomonas maltophilia* (formerly *Pseudomonas maltophilia*), a species rarely involved in human infections at that time [28]. Consequently, these enzymes were regarded as having limited or no clinical relevance. In addition, other chromosomally-encoded, species-specific serine carbapenemases, such as the SME (*Serratia marcescens* enzyme), were occasionally reported during the 1980s in Europe and the United States [29,30,31]. Clinical concern intensified in the following decades, when these enzymes began to emerge in more common clinical isolates (Enterobacterales, *P. aeruginosa*), particularly after their encoding genes were found to be located on plasmids, making them easily transferable to other microorganisms and causing outbreaks in many parts of the world.

At present, carbapenemases have been identified within each of the Ambler molecular classes A, B, and D, while class C β-lactamases are recognized as significant contributors to carbapenem resistance in GNB.

### 3.1. Class A Carbapenemases

Six epidemiologically important enzyme families belonging to this class of serine β-lactamases (SBLs) are capable of hydrolyzing carbapenems: SME (*Serratia marcescens* enzyme), NMC-A (non-metallo class A carbapenemase), IMI (imipenem-hydrolyzing β-lactamase), SFC (*Serratia fonticola* carbapenemase), KPC (*Klebsiella pneumoniae* carbapenemase) and certain enzymes of the GES (Guiana extended-spectrum enzyme) family. Although most members of the GES family are classified as ESBLs, specific point mutations have expanded their substrate specificity to include carbapenems (e.g., GES-2, GES-5) [32]. They all belong to subgroup 2f in the Bush–Jacoby functional β-lactamase classification, having carbapenems as preferred substrates, but being able to also hydrolyse all other β-lactam antibiotics, including penicillins, cephalosporins, and monobactams [33].

Enzymes belonging to the first four families are relatively uncommon and are chromosomally encoded, exhibiting a species-specific distribution, namely in *Enterobacter cloacae* complex (NMC-A and IMI) and *Serratia* spp. (SME and SFC) [34]. However, although uncommon, a limited number of outbreaks involving carbapenemases of the SME and IMI families have been documented [35,36,37].

In contrast, KPC and GES enzymes are usually acquired and are frequently associated with plasmids and other MGEs, rendering them the most clinically and epidemiologically relevant, particularly those belonging to the KPC family.

KPC enzymes were initially identified in the United States in 1996 in a *K. pneumoniae* isolate—from which they derive their name—and were first reported in the scientific literature in 2001 [38]. Since then, they have disseminated rapidly across multiple regions worldwide and have been implicated in major outbreaks of MDR GNB, especially KPC-2 and KPC-3. The localization of the *bla*KPC gene on plasmids, which facilitates efficient horizontal transfer among GNB, together with its predominant association with *K. pneumoniae*—one of the most frequent causative agents of HAIs, known for its exceptional capacity to both acquire and disseminate antimicrobial resistance determinants —accounts for KPC being regarded as the most clinically significant class A carbapenemase. Moreover, it is considered that KPC enzymes can occur in nearly all GNB, having been identified in multiple species within the order Enterobacterales (e.g., *Enterobacter* spp., *Salmonella* spp.), as well as among NF-GNB, including *P. aeruginosa* and *A. baumannii* [27,39,40].

First described in 2001 in a *P. aeruginosa* isolate from South Africa, carbapenemases belonging to the GES family are identified far less frequently than those of the KPC family, despite also being plasmid-encoded. Nevertheless, GES-type carbapenemases have been reported in multiple regions worldwide, including countries in Europe, Africa, South America, and Asia. Most reports involve sporadic cases [41], although limited outbreak events have also been documented, such as a small nosocomial cluster involving GES-2–producing *P. aeruginosa* in South Africa and the identification of GES-5 in *K. pneumoniae* isolates from six patients in Korea during the early 2000s [42,43].

### 3.2. Class B Carbapenemases

These carbapenemases are metalloenzymes (MBLs) distinguished by a remarkably broad substrate profile, encompassing all or nearly all β-lactam antibiotics (depending on the enzyme) with the exception of monobactams (aztreonam). On the basis of their structural features and active-site architecture, MBLs are subdivided into three subclasses: B1, B2, and B3. Enzymes in subclasses B1 and B3 coordinate two Zn^2+^ ions at the catalytic site and correspond functionally to subgroup 3a, exhibiting strong activity against carbapenems as well as penicillins and cephalosporins [33,44]. In contrast, subclass B2 carbapenemases achieve maximal catalytic activity when coordinated to a single Zn^2+^ ion, whereas binding of a second Zn^2+^ ion leads to enzymatic inhibition [41,45,46]. These enzymes are assigned to functional subgroup 3b and display a marked preference for carbapenems, with comparatively weak activity toward penicillins and cephalosporins [33,47].

Initially, as already mentioned, the only β-lactamases known to have carbapenems as substrates were MBLs, and the genes encoding them were located on the chromosomes of species that were not involved, or were only rarely involved, in human infectious pathology. Consequently, they were considered clinically insignificant [44]. This situation changed drastically, however, when the first plasmid-encoded MBLs belonging to the IMP (imipenemase) and VIM (Verona integron-encoded metallo-β-lactamase) families emerged during the 1990s in Japan and Italy, respectively. Initially identified in *P. aeruginosa* isolates, these MBLs may also be encountered in Enterobacterales species or other NF-GNB such as *A. baumannii*, particularly the enzymes from the IMP family [41,44,48]. Both families have subsequently disseminated internationally, although their prevalence remains strongly region-dependent. VIM enzymes are particularly important among CRPA in several regions, whereas IMP enzymes remain especially prevalent in parts of the Asia-Pacific regions. Both have been implicated in numerous healthcare-associated outbreaks [49,50,51].

In contrast, enzymes belonging to the NDM (New Delhi metallo-β-lactamase) family, first identified in Sweden in 2008 in *Escherichia coli* and *K. pneumoniae* strains isolated from a patient returning from India, and subsequently reported in the literature in 2009, achieved extensive worldwide dissemination across multiple species of GNB. They currently represent the most prevalent MBLs and are among the most frequently identified carbapenemases in recent years, particularly in Enterobacterales, but also in NF-GNB, including *P. aeruginosa* and *A. baumannii* [48,52]. Unlike *bla*IMP and *bla*VIM genes, which are integron-associated and may be inserted into either chromosomal or plasmid DNA of the bacteria, the genes encoding NDM production are frequently located on highly transmissible plasmids with a broad host range [44,48]. Furthermore, these plasmids often harbor additional resistance determinants against other classes of antibiotics, rendering infections caused by bacteria carrying these plasmids particularly difficult to treat [18,53], but at the same time indicating that the dissemination of carbapenem-resistance genes may be driven by selective pressures beyond those exerted solely by β-lactam antibiotics [27].

Another distinctive feature of NDM is its cellular localization, which differs substantially from that observed in other MBLs. Unlike most of these enzymes, which are soluble proteins localized within the periplasmic space, NDM is a lipoprotein anchored to the outer membrane of GNB. This particular membrane-associated localization substantially increases the stability of the enzyme under zinc-depleted conditions (which may occur at the infection site, as a result of the host immune response) and also facilitates its release through outer membrane vesicles (OMVs). Experimental studies have also shown that these vesicles may carry both the NDM enzyme, thereby conferring protection to neighboring bacterial cells against β-lactam antibiotics, and the corresponding *bla*NDM gene, thus providing a potential additional route for dissemination of carbapenem resistance, although the epidemiological importance of this mechanism remains uncertain [54,55,56].

IMP, VIM and NDM all belong to the B1 subclass. Due to their ease of transmission and dissemination, as well as the associated clinical and therapeutic implications, these enzymes are considered the most clinically and epidemiologically relevant MBLs. Other widely distributed acquired MBLs from this subclass include SPM (São Paulo metallo-β-lactamase), identified in a *P. aeruginosa* isolate from Brazil in 1997 and reported in the early 2000s [57]; GIM (German imipenemase) identified in Germany in 2002 also in a *P. aeruginosa* isolate and molecularly described in 2004 [58]; and SIM (Seoul imipenemase) identified in an *A. baumannii* isolate in Korea during 2003–2004 and reported in 2005 [59].

The remaining MBLs within subclasses B2 and B3 are mostly intrinsic, chromosomally encoded, species-specific carbapenemases, such as CphA (subclass B2) in *Aeromonas hydrophila*, as well as L1 and GOB-1 (both subclass B3) identified in *Stenotrophomonas maltophilia* and *Elizabethkingia meningoseptica*, respectively. Although these enzymes are generally chromosomally encoded and therefore considered non-transferable (with few exceptions), their clinical relevance has increased in recent years. This trend is largely attributable to the rising incidence of infections caused by their host bacteria. Notably, *Stenotrophomonas maltophilia*, a MDR microorganism, has emerged with increasing frequency as an opportunistic pathogen, particularly in immunocompromised patients [48].

### 3.3. Class D Carbapenemases

Several OXA-type serine β-lactamases, initially designated as oxacillinases because many of the earliest members had the ability to hydrolyse oxacillin and cloxacillin more efficiently than benzylpenicillin, have subsequently been demonstrated to exhibit hydrolytic activity against carbapenems. This highly heterogeneous group of enzymes, collectively known as carbapenem-hydrolysing class D β-lactamases (CHDLs), is classified within subgroup 2df according to the Bush–Jacoby functional classification.

The first CHDL was identified in 1985 in a MDR *A. baumannii* isolate recovered from the blood culture of a patient from Scotland and was subsequently characterized and described in the literature in 1993 as the ARI-1 enzyme (*Acinetobacter* resistant to imipenem), currently designated OXA-23 [60]. In 1995, it was further demonstrated that the gene encoding this enzyme was located on a large plasmid, highlighting its potential for horizontal dissemination [61]. Consequently, during the following years, clinical isolates of CRAB were increasingly reported in healthcare settings worldwide, harboring not only OXA-23, but also OXA-24/40, OXA-58, and subsequently, to a lesser extent, OXA-143 and OXA-235 carbapenemases. In addition to these acquired carbapenemases, OXA-51 and OXA-51-like enzymes were identified in all strains of *A. baumannii*, being intrinsically encoded at the chromosomal level [11,41].

Although initially identified predominantly in *A. baumannii*, where they constitute the most prevalent mechanism of carbapenem resistance, these carbapenemases have subsequently been detected in other NF-GNB, including OXA-50 in *P. aeruginosa*, as well as among members of Enterobacterales, such as OXA-23 in *Proteus mirabilis* and OXA-48 and OXA-48-like enzymes (e.g., OXA-162, OXA-181, OXA-204, and OXA-232, with OXA-181 and OXA-232 being among the most widely disseminated variants) in *K. pneumoniae*, *E. coli*, *Enterobacter cloacae*, and *Citrobacter freundii*, the latter representing the only OXA-type carbapenemases to have achieved widespread dissemination within this bacterial order [62,63,64].

OXA-48 was first identified in Turkey in 2001 in a clinical isolate of *K. pneumoniae* [65] and has subsequently disseminated extensively worldwide among members of Enterobacterales, being implicated in numerous healthcare-associated outbreaks globally. Although OXA-48 and OXA-48-like carbapenemases are predominantly associated with Enterobacterales, sporadic reports have described their detection in NF-GNB, particularly *P. aeruginosa* and, more exceptionally, *A. baumannii*, highlighting the capacity of these resistance determinants to disseminate beyond their traditional Enterobacterales hosts [66,67,68]. Among OXA-type carbapenemases, OXA-48 exhibits relatively greater carbapenem-hydrolyzing activity, although its catalytic efficiency remains inferior to that of many class A and class B carbapenemases. As a consequence, these carbapenemases frequently confer only low-level in vitro carbapenem resistance, rendering their detection particularly challenging and suggesting that the burden associated with these enzymes may be underestimated [69]. Furthermore, concerns regarding these enzymes are also related to their remarkable capacity to undergo minimal mutational changes that may substantially expand their hydrolytic spectrum [63]. Nevertheless, these characteristics, together with their plasmid-mediated dissemination, frequent co-expression with other β-lactamases, and association with additional carbapenem-resistance mechanisms, collectively contribute to the emergence of OXA-48-producing organisms as a major contemporary public health concern.

OXA enzymes exhibit several distinctive characteristics that differentiate them from the majority of other carbapenemases. They exhibit relatively low hydrolytic activity against carbapenems, a characteristic feature of class D β-lactamases, which generally display lower catalytic efficiency toward their substrates compared with β-lactamases belonging to other Ambler classes [33,41]. However, when *bla*OXA genes become associated with insertion sequences, as frequently observed in *A. baumannii*, this association may result in the overexpression of these genes, thereby leading to increased resistance to carbapenems. Simultaneously, insertion sequences may facilitate the horizontal transfer of OXA genes, consequently contributing to their dissemination among distinct bacterial strains and across diverse geographical regions worldwide [70].

Furthermore, similarly to the mechanism previously described for NDM enzymes, experimental studies have demonstrated that OXA-23 and OXA-24/40 are outer membrane-associated proteins in *A. baumannii*, in contrast to OXA-48 in Enterobacterales, which is a soluble protein localized within the periplasmic space of these bacteria. As a consequence, OXA-23 and OXA-24/40 may be incorporated into OMVs released by the bacterium, thereby conferring a protective effect on neighboring bacterial populations [71].

Nevertheless, when expressed in microorganisms characterized by impaired outer membrane permeability, such as *A. baumannii*, which intrinsically lacks several major porins characteristic of Enterobacterales, including OmpC and OmpF, or in the presence of additional resistance mechanisms such as efflux pumps—frequently encountered in NF-GNB—these enzymes are capable of exerting clinically significant hydrolytic activity against carbapenems [11].

With regard to Ambler **class C β-lactamases**, although these enzymes are not traditionally classified as carbapenemases, such β-lactamases may contribute substantially to carbapenem resistance either through overproduction of AmpC β-lactamases as a result of gene overexpression, or through a combination of this mechanism with either loss, reduced expression, or structural alteration of major OMPs, leading to poor penetration of the antibiotic into the periplasmic space, thereby preventing it from reaching its target site and exerting its antimicrobial activity, or with increased efflux pump activity that expels the antibiotic before it can reach its target. However, rare class C enzymes or variants with demonstrable carbapenem-hydrolysing activity (e.g., CMY-10, ADC-68) have also been described [32,72].

## 4. Therapeutic Options Available for CR-GNB Infections

This section reviews currently available antimicrobial agents with activity against CR-GNB that have received regulatory approval from the FDA, the EMA, or both. These include newer BL/BLI combinations as well as other β-lactam and non-β-lactam agents introduced predominantly during the past decade. Ceftolozane-tazobactam, initially approved by the FDA in 2014, is also included because of its important carbapenem-sparing role, particularly in infections caused by MDR *P. aeruginosa*. For each agent, the following aspects are discussed: spectrum of activity against clinically relevant CR-GNB and carbapenemase types, approved clinical indications, major limitations, and currently described mechanisms of resistance.

Because resistance identified under different experimental or clinical circumstances does not carry the same clinical significance, evidence regarding resistance is categorized according to the context in which it has been demonstrated: baseline resistance, laboratory-derived resistance, and treatment-emergent resistance.

Baseline resistance refers to resistance detected among clinical isolates before exposure to the antimicrobial agent, including isolates identified in surveillance collections or pretreatment clinical specimens. Such resistance may reflect pre-existing resistance determinants, including mechanisms selected by previous exposure to other antimicrobial agents and, in some instances, cross-resistance between structurally or mechanistically related compounds. Surveillance or baseline susceptibility is particularly relevant for determining whether a newly introduced agent can be used reliably for empirical therapy within a given clinical and epidemiological context [73,74].

In vitro (laboratory-derived) resistance refers to resistance demonstrated under controlled experimental conditions. This includes mutants selected through serial passages or other forms of repeated antimicrobial exposure, as well as mechanistic studies employing site-directed mutagenesis, allelic replacement, or expression of resistance determinants or enzyme variants in defined genetic backgrounds [75,76,77,78]. These approaches are particularly valuable for identifying resistance pathways and establishing causal relationships between specific genetic alterations and reduced antimicrobial susceptibility. However, experimentally demonstrated mechanisms do not necessarily indicate that the same phenotype will readily emerge under clinically achievable drug exposures or during treatment in patients.

Treatment-emergent resistance refers to the development of reduced susceptibility or resistance in isolates recovered from the same patient during or following therapy with the antimicrobial agent concerned, relative to a susceptible or more susceptible pretreatment isolate [79]. When supported by appropriate microbiological and, where available, molecular evidence demonstrating persistence or evolution of the infecting strain, this represents the most direct evidence that resistance may emerge under clinical antimicrobial exposure. Such observations are particularly relevant in persistent, recurrent, or relapsing infections and support repeat antimicrobial susceptibility testing (AST) of subsequently recovered isolates when resistance emergence is clinically suspected [73].

To facilitate comparison among therapeutic options, the information discussed in this section is summarized in two tables. Table 1 compares the activity of currently available agents according to the major carbapenemases and most clinically relevant CR-GNB pathogens, together with their approved indications and principal clinical limitations. Table 2 focuses on resistance to newer therapeutic agents and summarizes the therapeutic agents, the relevant organisms, the resistance mechanisms demonstrated to date, the level and context of supporting evidence, and the potential clinical implications of the reported resistance mechanisms.

### 4.1. New BL/BLI Combinations

In the context of the increasingly widespread dissemination of carbapenemase-producing CR-GNB in contemporary clinical practice, coupled with the limited development of novel antibacterial agents over recent decades, the need for broad-spectrum β-lactamase inhibitors (BLIs) has become increasingly pressing. Consequently, following a development gap of more than two decades, two novel classes of BLIs have emerged: diazabicyclooctanes (DBOs) and boronic acid derivatives. These inhibitors differ substantially from first-generation BLIs, both structurally and mechanistically. Structurally, they are characterized by a diazabicyclooctane or boronic acid core rather than a β-lactam scaffold. Mechanistically, they inhibit β-lactamases through the formation of a reversible covalent enzyme–inhibitor complex during enzyme inactivation, in contrast to the irreversible “suicidal” inhibition exhibited by first-generation BLIs.

#### 4.1.1. Clinically Approved DBO BLIs and Their β-Lactam Combinations

The first next-generation BLI to be introduced into clinical practice was **avibactam**, a member of the DBO class. Compared with first-generation BLIs, avibactam exhibits a substantially broader inhibitory spectrum, encompassing the majority of clinically relevant Ambler class A β-lactamases, including TEM- and SHV-type penicillinases, ESBLs of the TEM, SHV, and CTX-M families, and, most importantly, KPC carbapenemases. In addition, avibactam demonstrates potent inhibitory activity against Ambler class C cephalosporinases (AmpC β-lactamases) and selected class D β-lactamases, particularly OXA-48-like carbapenemases [80].

Avibactam is currently marketed in combination with ceftazidime, a third-generation cephalosporin, and, more recently, with aztreonam.

**Ceftazidime–avibactam** was approved by the FDA in 2015 for the treatment of complicated urinary tract infections (cUTIs) and complicated intra-abdominal infections (cIAIs) in combination with metronidazole, with approval subsequently extended in 2018 to include hospital-acquired pneumonia (HAP) and ventilator-associated pneumonia (VAP). The European Medicines Agency (EMA) granted approval for the same indications in 2016 [81]. Its antimicrobial activity is limited against GP organisms and anaerobes, with the lack of anaerobic coverage explaining the need for co-administration of metronidazole in the treatment of cIAI [82,83,84].

Owing to avibactam’s potent inhibition of serine β-lactamases, CAZ–AVI has become an important therapeutic option for infections caused by KPC- and OXA-48-like-producing Enterobacterales, exhibiting in vitro activity against approximately 96–99% of such isolates [85]. Furthermore, the combination retains activity against β-lactam-resistant *P. aeruginosa* strains, including isolates characterized by AmpC overexpression and OprD porin deficiency, although its antipseudomonal activity is generally considered inferior to that of ceftolozane–tazobactam [40] (Table 1).

Regarding baseline resistance, despite its broad spectrum, CAZ–AVI lacks activity against MBL-producing organisms, including those producing enzymes belonging to the NDM, VIM, and IMP families, owing to the inability of avibactam to inhibit these β-lactamases [83,86]. Similarly, the combination demonstrates limited to no activity against *A. baumannii* [86].

Furthermore, although CAZ–AVI has represented a major therapeutic advance in the management of infections caused by MDR GN pathogens, particularly carbapenemase-producing Enterobacterales (CPE), with numerous studies demonstrating significantly improved clinical outcomes compared with previously available treatment options (mainly colistin-based combination therapy, but also aminoglycosides, tigecycline, fosfomycin) [87,88,89,90,91,92,93,94,95], its long-term effectiveness is increasingly threatened by the emergence of resistance to this agent [96]. As the first novel BL/BLI combination capable of successfully treating some of the most challenging infections while exhibiting a more favorable safety profile than previously employed therapeutic regimens—particularly by avoiding the nephrotoxicity and neurotoxicity commonly associated with polymyxins (e.g., colistin) and aminoglycosides—CAZ-AVI has rapidly become an essential component of the therapeutic armamentarium against MDR GN pathogens, particularly KPC producing *K. pneumoniae*. Concurrently, the incidence of infections caused by carbapenem-resistant organisms (CROs) has increased at an alarming rate over recent years, leading to a substantial expansion in the clinical use of CAZ-AVI. Consequently, and in accordance with the evolutionary adaptations that enable bacteria to survive under antimicrobial selective pressure, an increasing number of reports have documented the emergence of resistance to CAZ-AVI.

As predicted by early in vitro studies demonstrating that CAZ–AVI resistance could readily emerge in KPC-producing Enterobacterales following antibiotic exposure through the selection of resistant mutants, particularly among KPC-3-producing isolates [77], clinical reports published shortly after the approval of CAZ–AVI confirmed these concerns. The most frequently described mechanism of resistance emerging during CAZ–AVI therapy involves amino acid substitutions, insertions, or deletions within KPC carbapenemases, particularly KPC-2 and KPC-3, with numerous KPC variants associated with CAZ-AVI resistance having subsequently been identified, most of them in the last few years [97]. These structural alterations reduce the inhibitory activity of avibactam while simultaneously increasing the affinity of the enzyme for ceftazidime, thereby conferring resistance to CAZ–AVI [86,97,98,99,100,101]. Importantly, the emergence of *bla*KPC-3 mutations during CAZ-AVI therapy was demonstrated directly in longitudinal clinical isolates by Shields et al., providing clinical evidence for treatment-emergent resistance under CAZ-AVI selective pressure [99].

Interestingly, certain KPC variants associated with CAZ–AVI resistance have also been shown to confer a transient partial or complete restoration of susceptibility to carbapenems, resulting in an ESBL-like phenotype. This phenomenon, commonly referred to as the “see-saw effect”, reflects an evolutionary trade-off in which structural alterations within the KPC enzyme reduce its carbapenemase activity while simultaneously increasing its hydrolytic efficiency toward ceftazidime and decreasing its susceptibility to inhibition by avibactam [98,99,100,101]. Consequently, these KPC variants exhibit reduced carbapenem hydrolysis despite conferring resistance to CAZ–AVI. Although these observations raise the possibility that carbapenems may regain therapeutic activity against selected CAZ–AVI-resistant isolates, this restored susceptibility is frequently unstable. In vitro studies have demonstrated that exposure to carbapenems of CAZ-AVI-resistant, meropenem-susceptible *K. pneumoniae* isolates carrying KPC-3 variant enzymes can rapidly select for resistant subpopulations through the acquisition of compensatory adaptive mechanisms, most commonly involving alterations in OMPs, that restore high-level carbapenem resistance, while maintaining CAZ-AVI resistance as well [102]. Therefore, the apparent reversion to carbapenem susceptibility should be interpreted with caution and confirmed by repeated susceptibility testing when clinically relevant.

Furthermore, the emergence of these KPC variants complicates the laboratory identification of KPC-producing microorganisms, with potential therapeutic implications that may ultimately lead to unfavorable clinical outcomes. On one hand, certain diagnostic assays, such as immunochromatographic tests, may yield false-negative results likely due to structural alterations affecting the antibody-binding epitopes within the carbapenemase enzyme [103]. On the other hand, in KPC variants exhibiting the so-called “see-saw effect”, some phenotypic assays may incorrectly classify isolates as displaying an ESBL phenotype rather than identifying them as CRE [97].

In addition to the emergence of KPC variants, in vitro and molecular evidence indicates that several other mechanisms may contribute to reduced CAZ–AVI susceptibility or resistance. These include reduced outer membrane permeability resulting from alterations in OmpK35 and OmpK36 porins, increased *bla*KPC-2 and *bla*KPC-3 gene copy number or overexpression, upregulation of efflux pumps, and the co-production of and/or mutations in genes encoding additional β-lactamases (such as SHV, CTX-M, VEB, AmpC). These mechanisms frequently act in concert, contributing to higher levels of resistance and complicating therapeutic management [86,104].

Because CAZ–AVI lacks activity against MBLs, and given the widespread dissemination of these enzymes in recent years, which has established them as one of the most significant challenges in the management of MDR GNB infections, CAZ–AVI has been used in combination with aztreonam for the treatment of infections caused by MBL-producing isolates. This therapeutic strategy is based on the unique stability of aztreonam to hydrolysis by MBLs, making it the only β-lactam agent that is not a substrate for these enzymes. More recently, in response to the critical need for effective therapeutic options against MBL-producing pathogens, particularly NDM-producing CRE, a fixed combination of aztreonam and avibactam, **aztreonam–avibactam** (ATM–AVI), has been developed [23,86]. This fixed-dose formulation simplifies the administration of an effective therapeutic regimen while eliminating unnecessary exposure to ceftazidime in clinical settings where its antibacterial activity provides no additional therapeutic benefit [105].

ATM–AVI received approval from the EMA in April 2024 for the treatment of cIAIs, cUTIs, including pyelonephritis, HAP, VAP, and infections caused by aerobic GNB in patients with limited therapeutic options [23,105]. Subsequently, in February 2025, the FDA approved ATM–AVI, in combination with metronidazole, for the treatment of cIAIs in adult patients, including those with limited or no alternative treatment options [106].

The therapeutic value of ATM–AVI derives from the complementary activities of its two components. Aztreonam is intrinsically resistant to hydrolysis by MBLs, whereas avibactam protects aztreonam from degradation by co-produced SBLs, including Ambler class A enzymes (such as KPC carbapenemases and ESBLs), class C enzymes (AmpC β-lactamases), and certain class D enzymes, particularly OXA-48-like β-lactamases [23,105] (Table 1).

However, ATM–AVI lacks activity against GP bacteria, anaerobic organisms, and *Acinetobacter* spp.; consequently, additional antimicrobial agents may be required in the setting of polymicrobial infections [23,105]. Furthermore, ATM–AVI is unlikely to represent an optimal therapeutic option for infections caused by MBL-producing *P. aeruginosa*. In vitro studies have demonstrated limited activity of this combination against such isolates, largely reflecting the multifactorial nature of β-lactam resistance in *P. aeruginosa*. In addition to MBL production, these organisms frequently harbor concurrent resistance mechanisms, including reduced outer membrane permeability, overexpression of efflux pumps, and the co-production or hyperexpression of additional β-lactamases, such as the chromosomally encoded AmpC β-lactamase (PDC) or uncommon carbapenemases that are not inhibited by avibactam. The coexistence of these resistance determinants may substantially compromise the antibacterial activity of ATM–AVI [105,107,108,109].

Notably, baseline resistance to ATM–AVI was documented even before its widespread clinical introduction, suggesting that it primarily reflects pre-existing resistance mechanisms, possibly selected through prior exposure to β-lactam-based therapeutic regimens, including novel BL/BLI combinations already widely used in clinical practice [110]. To date, resistance has been described predominantly among NDM-producing *E. coli* isolates belonging to some internationally disseminated high-risk sequence types, such as ST405, ST410, ST167, or ST361 [110,111,112,113,114]. The principal mechanism involves characteristic amino acid insertions in PBP3, the primary target of aztreonam, together with the co-production of additional acquired β-lactamases, particularly AmpC enzymes belonging to the CMY-2 family, mainly CMY-42 [111,112,115], but also CMY-145 [112,113] and, less commonly, other ESBLs such as CTX-M-15 [112,116,117]. In addition, decreased outer membrane permeability resulting from alterations in the major porins OmpC and OmpF, together with occasional overexpression of efflux pumps, may further contribute to reduced susceptibility to ATM–AVI in these isolates [114].

The fact that the predominant mechanism of ATM–AVI resistance in NDM-producing *E. coli* involves structural alterations in PBP3 has major therapeutic implications. Because PBP3 constitutes the target not only of aztreonam but also of other β-lactams, including cefepime and cefiderocol, these alterations may reduce the activity of additional agents active against MBL-producing pathogens [118,119,120]. This is of particular concern given the already limited therapeutic armamentarium available for such infections, potentially compromising the efficacy of both cefiderocol, currently in clinical use, and the investigational combination cefepime–taniborbactam, which has completed Phase III clinical development and is currently undergoing regulatory review.

In addition to this baseline resistance, treatment-emergent increases in ATM-AVI resistance have also been documented clinically. Haidar et al. reported the selection of high-level ATM-AVI resistance following treatment with AZT plus CAZ-AVI in an NDM-5-producing *E.coli* isolate that already exhibited reduced susceptibility to ATM-AVI at baseline [120].

Although reported less frequently, resistance has also been described in other clinically important Enterobacterales, particularly *K. pneumoniae*, where it is primarily driven by the combined contribution of multiple resistance mechanisms, including overproduction of KPC enzymes or production of specific KPC variants, CMY-type AmpC β-lactamases, alterations in the major outer membrane porins OmpK35 and OmpK36, and, in some isolates, efflux pump overexpression [112,121,122,123,124,125]. Both in vitro and treatment-emergent evidence support resistance in these species. In vitro selection experiments have demonstrated that ATM-AVI resistance can emerge under antimicrobial exposure in *K. pneumoniae* [122,123], while in vivo emergence of ATM-AVI cross-resistance has also been reported in a patient with KPC-producing *K. pneumoniae* following cefiderocol-based treatment [124].

Overall, the available baseline, in vitro, and treatment-emergent evidence indicates that ATM–AVI resistance is typically multifactorial, resulting from the cumulative effects of target-site modifications, β-lactamase production, impaired outer membrane permeability, and active efflux rather than from a single resistance determinant.

**Relebactam** is the second DBO BLI introduced into clinical practice, following avibactam, to which it is structurally related [126]. Although relebactam lacks intrinsic antibacterial activity, it effectively inhibits a broad range of Ambler class A and class C β-lactamases (including ESBLs and, more importantly, KPC and PDCs, respectively) [127], while exhibiting limited or no activity against selected class D carbapenemases and no activity against MBLs [86]. Furthermore, its distinctive chemical structure renders it a poor substrate for efflux pumps, a common mechanism of antibiotic resistance among NF-GNB, particularly *P. aeruginosa* [128].

Clinically, relebactam is available in combination with imipenem-cilastatin as the fixed-dose formulation **imipenem–cilastatin–relebactam** (IMI–REL).

IMI–REL represents a novel BL/BLI combination with enhanced activity against carbapenem-resistant non-Morganellaceae Enterobacterales (CR-NME) and DTR *P. aeruginosa* [127]. It has no in vitro activity against strains of *Morganella* spp., *Providencia* spp., and *Proteus* spp., and therefore its use as empirical therapy is not recommended in healthcare settings with a high prevalence of these pathogens [86]. Moreover, the antimicrobial activity of IMI–REL against *Acinetobacter* spp. (including CRAB) is limited, rendering the combination largely ineffective against these organisms [127] (Table 1).

The combination received approval from the FDA in 2019 for the treatment of adults with cUTIs and cIAIs who have limited or no alternative therapeutic options. In 2020, its indications were expanded to include HAP and VAP. During the same year, the EMA approved IMI–REL for the treatment of aerobic GN bacterial infections in adults with limited therapeutic alternatives [126,129].

Within the European Union, IMI-REL is currently indicated for the treatment of HAP and VAP, bacteremia associated with or suspected to be associated with these infections, and other aerobic GN bacterial infections in patients for whom alternative treatment options are limited. Furthermore, owing to the presence of imipenem, this combination retains activity against certain GP organisms, including ampicillin-susceptible enterococci, as well as against selected GN anaerobes. Consequently, unlike other recently introduced BL/BLI combinations, IMI-REL may represent a particularly attractive therapeutic option for polymicrobial infections in which these organisms coexist with CR-NME and/or DTR *P. aeruginosa* [127].

IMI-REL generally retains activity against DTR *P. aeruginosa* isolates exhibiting resistance mediated by OprD loss or the overexpression of major efflux systems, including MexAB-OprM, MexCD-OprJ, and MexXY [117,118,120]. However, baseline resistance to IMI-REL in *P. aeruginosa* may be found in strains that produce certain class A β-lactamases, such as VEB ESBLs and GES type carbapenemases, class B MBLs (VIM, IMP, NDM) or certain class D OXA-type carbapenemases [127,128,130].

In CR-NME, baseline reduced susceptibility or resistance to IMI-REL has been reported among isolates producing OXA-48-like carbapenemases or MBLs, as well as *Serratia marcescens* producing SME carbapenemase [127]. Baseline resistance has also been identified among KPC-producing *K. pneumoniae* strains. In a retrospective analysis of isolates collected before the clinical introduction of IMI-REL, resistant KPC-producing *K. pneumoniae* harboured OmpK36 alterations in conjunction with KPC production, supporting the contribution of impaired outer-membrane permeability to resistance [131].

In vitro laboratory-selected resistance has also been demonstrated in KPC-producing *K. pneumoniae*. Following serial exposure to increasing concentrations of IMI-REL, resistance was associated with OmpK36 disruption and modifications of KPC enzymes, together with additional strain-specific mutations [132]. These findings indicate that the accumulation of permeability defects and alterations affecting the interaction of relebactam with KPC may promote the development of IMI-REL resistance under selective pressure [132].

With regard to clinically evolved resistance, increased *bla*KPC copy number together with disruption of OmpK35 and OmpK36 has been reported in a KPC-producing *K. pneumoniae* isolate [133]. Importantly, however, this resistance did not emerge during IMI-REL therapy, but rather it developed in vivo, in a patient exposed to multiple antimicrobial regimens based on CAZ-AVI and MEM-VAB and was associated with cross-resistance to IMI-REL [133]. Therefore, this report provides evidence of clinically evolved IMI-REL resistance under exposure to other novel BL/BLI combinations rather than direct treatment-emergent resistance during IMI-REL therapy.

Notably, IMI-REL has also demonstrated in vitro activity against isolates harboring KPC-3 or PDC variants associated with resistance to other novel BL/BLIs, such as CAZ-AVI or CTL-TZB [127,134].

**Durlobactam** is a DBO-based non-β-lactam BLI specifically developed for the treatment of infections caused by the *Acinetobacter baumannii–calcoaceticus* complex (ABC), including carbapenem-resistant isolates. This novel BLI is co-formulated with sulbactam, a first-generation BLI that retains intrinsic antibacterial activity, particularly against *A. baumannii*, owing to its β-lactam structure and affinity for PBPs [23,135].

**Sulbactam–durlobactam** (SUL–DUR) is indicated for the treatment of HAP and VAP caused by ABC, particularly CRAB [23].

SUL–DUR was approved by the FDA in 2023 for use in adult patients; however, it has not yet received approval from the EMA and is therefore currently unavailable within the European Union [23,135].

Despite sulbactam’s intrinsic antibacterial activity against *Acinetobacter* spp. through its binding to PBPs, its efficacy is frequently compromised by hydrolysis mediated by β-lactamases produced by most ABC isolates. Durlobactam exhibits potent inhibitory activity against a broad range of Ambler class A, C, and D serine β-lactamases, including OXA-type enzymes, thereby protecting sulbactam from enzymatic degradation and restoring its activity against CRAB strains [135,136] (Table 1).

Although resistance to SUL-DUR among ABC, including CRAB isolates, remains relatively uncommon, it has already been documented. Owing to the recent introduction of this combination into clinical practice, current knowledge regarding resistance mechanisms and prevalence is derived predominantly from in vitro studies, antimicrobial surveillance programs, and very few clinical studies and case reports [137,138,139,140,141,142].

Regarding baseline resistance, SUL–DUR lacks activity against ABC isolates harboring Ambler class B MBLs, particularly NDM enzymes, which are not inhibited by durlobactam and are being detected with increasing frequency among clinical isolates, particularly in certain regions of the world, and against which the combination remains uniformly ineffective [136,137,143,144]. In addition, alterations in PBP3, the primary target of sulbactam, represent a major mechanism of baseline SUL–DUR resistance or reduced susceptibility in ABC. The impact of these target-site modifications is often amplified when combined with other resistance determinants, such as β-lactamase overproduction or efflux pump overexpression [137,138,140,142,144].

In vitro laboratory-selected SUL-DUR resistance has also been associated predominantly with alterations in PBP3, which reduce the affinity of sulbactam for its target. Additional alterations affecting efflux systems may further increase SUL-DUR MICs, particularly when occurring in combination with PBP3 modifications [137,138,139]. These findings are consistent with studies of clinical isolates in which PBP3 substitutions constitute a major molecular determinant of SUL-DUR non-susceptibility [137,138].

In contrast to other recently introduced BL/BLI combinations that have been in clinical use for a longer period and for which the emergence of resistance during therapy has been well documented, such as CAZ-AVI, reports of treatment-emergent resistance to SUL-DUR remain exceedingly rare. In the phase 3 ATTACK trial, resistance emerged during SUL-DUR treatment in one patient whose baseline isolate was susceptible to this combination. The baseline isolate harbored the PBP3 A515V variant, whereas subsequent non-susceptible isolates acquired an additional substitution in the AdeJ, a component of the AdeIJK efflux system. Because PBP3 A515V alone appears insufficient to confer resistance and durlobactam is a substrate of AdeIJK, the combined effect of reduced sulbactam target affinity and enhanced durlobactam efflux was considered the most likely mechanism underlying treatment-emergent resistance in this case [140]. In a separate longitudinal clinical report, SUL-DUR non-susceptibility was associated with the acquisition of a PBP3 H370Y substitution together with an AdeJ mutation [141]. However, these alterations arose before SUL-DUR administration, following exposure to several other β-lactam agents. Therefore, this case represents clinically evolved pre-treatment resistance rather than treatment-emergent resistance to SUL-DUR [141].

**Zidebactam** is a novel DBO-derived compound with a distinctive dual mechanism of action that differentiates it from previously introduced BLIs from this class. In addition to its β-lactamase inhibitory activity, particularly against Ambler class A and class C, unlike other DBO BLIs currently in use, zidebactam possesses intrinsic antibacterial activity mediated by high-affinity binding to PBP2 [145,146,147]. It is currently used in combination with cefepime, a fourth-generation cephalosporin whose principal target is PBP3, being active against a broad range of GN pathogens, including organisms producing AmpC β-lactamases [148,149,150]. The combination thus provides complementary PBP targeting, with cefepime predominantly inhibiting PBP3 and zidebactam additionally targeting PBP2. This complementary mechanism, together with inhibition of relevant β-lactamases and consequent protection of cefepime from enzymatic hydrolysis, underlies the enhanced antibacterial activity of this BL/BLI combination. Accordingly, zidebactam has been described as a β-lactam enhancer (BLE), rather than solely as a BLI [147,151,152].

**Cefepime/zidebactam** (CFP/ZID) is a novel BL/BLE combination that has recently entered clinical practice. In May 2026, the FDA approved CFP/ZID for the treatment of adult patients with cUTIs, including pyelonephritis, caused by designated susceptible microorganisms [24]. At present, CFP/ZID has not yet received marketing authorization from the EMA.

The complementary mechanisms of its two components confer a broad spectrum of in vitro activity against MDR GN pathogens. Studies have demonstrated activity against Enterobacterales and *P. aeruginosa*, including isolates producing KPC, OXA-48-like and MBLs [152,153,154,155,156], even in multiple carbapenemase-producers [157,158,159]. Of note, the CFP/ZID activity against MBL-producing organisms is not attributable to direct inhibition of MBLs by zidebactam, but rather to the BLE effect of zidebactam and its complementary PBP2 targeting in combination with cefepime-mediated PBP3 inhibition [147] (Table 1).

Importantly, CFP/ZID may retain activity against isolates resistant to several other recently introduced BL/BLI combinations. This has been particularly demonstrated among *P. aeruginosa* isolates resistant to CTL/TZB and CAZ/AVI, as well as in selected Enterobacterales isolates resistant to CAZ/AVI and ATM/AVI [118,153,160,161,162].

CFP/ZID has also demonstrated activity against CR *K. pneumoniae* carrying defective OmpK35 and OmpK36 porins, including isolates in which these permeability defects coexist with carbapenemase production [163]. In the cited collection, alterations or loss of these major porins, which can contribute to carbapenem resistance by reducing outer-membrane permeability, did not substantially compromise CFP/ZID activity.

In contrast, activity against CRAB has been more variable than that observed against Enterobacterales and *P. aeruginosa*. Earlier studies reported limited overall activity against *A. baumannii*, although lower MICs were observed among some isolates carrying acquired OXA-type carbapenemases, whereas MBL-producing isolates generally exhibited higher MICs [143,146,154,155]. More recent data, however, suggest that CFP/ZID may retain substantial in vitro activity against OXA-23-producing CRAB, including isolates resistant to SUL/DUR [164]. Nevertheless, given the small and geographically restricted isolate collection, comprising only 21 isolates from two Italian centres, these findings require confirmation in larger, geographically diverse studies, in order to better define its potential role in the treatment of CRAB infections.

Baseline resistance to CFP/ZID is generally associated with the co-existence of multiple resistance mechanisms in both Enterobacterales and *P. aeruginosa*. In Enterobacterales, alterations in PBP2 appear to be among the principal mechanisms contributing to reduced susceptibility or resistance to this combination [165,166,167]. In contrast, several studies have shown that alterations in PBP3 do not substantially compromise the activity of CFP/ZID, even in the presence of carbapenemases and other β-lactamases [118,165]. In addition, although CFP/ZID retains activity against several KPC variants, resistance has been observed in isolates producing certain KPC variants harboring mutations within the 270-loop region [168]. Furthermore, despite the generally preserved activity of CFP/ZID against MBL-producing Enterobacterales, resistance has been reported among some MBL-producing isolates, including those producing NDM-5 and NDM-14, particularly when MBL production is accompanied by other resistance mechanisms, such as impaired outer-membrane permeability resulting from alterations in major porins or PBP alterations [166,167,169].

With regard to *P. aeruginosa*, several studies have indicated that the accumulation of multiple resistance determinants, rather than a single mechanism, may be required to substantially impair CFP/ZID activity. Baseline resistance or reduced susceptibility to CFP/ZID in this pathogen has been associated primarily with efflux-pump overexpression, while the production of certain MBLs, especially NDM enzymes, may further compromise activity when occurring in combination with other resistance determinants [170,171].

Laboratory selection experiments further support the requirement for multiple resistance mechanisms to substantially impair CFP/ZID activity in *P. aeruginosa*. Resistance selected during serial in vitro exposure has been associated with alterations in its PBP targets, particularly PBP2 and PBP3, together with mutations leading to increased activity of efflux systems, particularly MexAB-OprM [172,173]. In one study, resistance to CFP/ZID appeared after the accumulation of multiple mutations affecting this efflux system and its regulators, together with PBP2 and PBP3 mutations [173], whereas another in vitro passaging study identified mutations in the gene encoding PBP2 in CFP/ZID-resistant derivatives of a CRPA clinical isolate [172].

Treatment-emergent resistance to CFP-ZID has recently been reported in a patient with *P. aeruginosa* pneumonia, in whom the CFP/ZID MIC increased during therapy and was associated with clinical failure. Whole-genome sequencing of longitudinal isolates identified newly acquired mutations affecting the MexAB-OprM efflux system, suggesting that increased efflux contributed to the emergence of resistance. These alterations were also associated with cross-resistance to CAZ-AVI and IMI-REL [174].

#### 4.1.2. Clinically Approved Boronic Acid Derivatives and Their β-Lactam Combinations

**Vaborbactam** is a non-β-lactam BLI whose structure is based on a cyclic boronic acid pharmacophore [86,126]. To date, it remains the only boronic acid-based BLI approved for clinical use. It is active against Ambler class A β-lactamases, including KPC-type carbapenemases as well as TEM, SHV, and CTX-M enzymes, and also exhibits activity against class C β-lactamases (AmpC). However, unlike avibactam, vaborbactam lacks inhibitory activity against Ambler class D carbapenemases, such as OXA-48-like enzymes. It is also ineffective against class B MBLs, including NDM, VIM, and IMP [81,86,126]. It has been developed and approved for clinical use in combination with meropenem as the fixed-dose BL/BLI combination meropenem–vaborbactam.

**Meropenem-vaborbactam** (MEM-VAB) received approval from the FDA in 2017 for the treatment of cUTIs in adults. Subsequently, in 2018, it was approved by the EMA for the treatment of cUTIs, including pyelonephritis, as well as cIAIs, HAP, VAP, and bacteremia associated with these infections. Within the European Union, the combination is also indicated for the treatment of infections caused by aerobic GN bacteria in adult patients with limited therapeutic alternatives [126,175]. In addition, it has been employed off-label for the management of other infections caused by non-CR GNB [81].

Although highly effective against infections caused by KPC-producing Enterobacterales, vaborbactam exhibits limited efficacy against CRPA [81,86] and lacks activity against *A. baumannii* [86] (Table 1).

Despite its relatively recent introduction into clinical practice, emerging resistance to MEM-VAB has been increasingly reported. Baseline resistance among KPC-producing *K. pneumoniae* has been associated with alterations affecting the expression or structure of the outer membrane porins OmpK35 and OmpK36. In particular, OmpK35 disruption combined with OmpK36 alterations, including insertions within the OmpK36 channel, has been associated with substantial reductions in MEM/VAB susceptibility, thereby compromising its clinical effectiveness [81,86].

In vitro-selected resistance has similarly been associated predominantly with impaired outer-membrane permeability. Laboratory-selected KPC-producing *K. pneumoniae* mutants have developed mutations or loss of OmpK36, frequently in backgrounds with already dysfunctional OmpK35. Increased KPC production secondary to increased *bla*KPC copy number has also been observed, particularly in association with porin alterations [78,81,86].

Although uncommon, treatment-emergent resistance during MEM-VAB therapy has also been reported. In a clinical study, recurrent infection caused by a MEM-VAB-nonsusceptible *K. pneumoniae* isolate with mutated OmpK36 developed in a patient after treatment with MEM-VAB [176]. Thus, unlike resistance to CAZ-AVI, which may involve structural changes in KPC enzymes, resistance to MEM-VAB appears to be driven predominantly by reduced outer-membrane permeability, sometimes accompanied by increased KPC production [78,81,86,176].

**Ceftolozane–tazobactam** (CTL-TZB) is a combination of a novel antipseudomonal cephalosporin and tazobactam, a well-established BLI.

Tazobactam, one of the first-generation BLIs, exhibits activity primarily against selected Ambler class A β-lactamases, including TEM, SHV, and CTX-M enzymes, but lacks inhibitory activity against KPC carbapenemases, class B MBLs, and class D OXA-48-like enzymes. Its activity against class C β-lactamases (AmpC) is limited and generally requires high concentrations [83].

Ceftolozane possesses potent antipseudomonal activity, attributable to its enhanced affinity for key PBPs (PBP1b, PBP1c, PBP3), stability against the chromosomal AmpC β-lactamases of *P. aeruginosa*, reduced susceptibility to efflux-mediated resistance, and activity that is largely independent of the OprD porin [83,177]. These characteristics make CTL–TZB a valuable therapeutic option for infections caused by MDR *P. aeruginosa*, including strains characterized by efflux pump overexpression or porin loss (non-CP CR strains), while maintaining lower MICs than many other antipseudomonal β-lactams [81,177] (Table 1).

The combination was approved by the FDA in 2014 for the treatment of cIAIs and cUTIs. In 2019, its indications were expanded to include HAP and VAP. The EMA has approved ceftolozane–tazobactam for the same indications [81].

Despite its potent activity against *P. aeruginosa*, CTL–TZB exhibits limited or no activity against other NF-GNB, including *Acinetobacter* spp. and *Stenotrophomonas* spp., which are generally resistant to this combination [83]. In addition, baseline resistance may also occur in carbapenemase-producing isolates or strains producing other β-lactamases that are not inhibited by tazobactam [81,178].

Although CTL–TZB demonstrates a relatively low propensity for resistance selection, with mutant prevention concentrations (MPCs) among the lowest reported for β-lactam agents [177], in vitro selection experiments in *P. aeruginosa* have demonstrated the development of high-level resistance through multiple mutations leading to overexpression and structural modifications of the chromosomal AmpC cephalosporinase (*Pseudomonas*-derived cephalosporinase, PDC) [166].

The emergence of resistance during CTL-TZB therapy has been documented as well. In *P. aeruginosa*, treatment-emergent resistance has been associated predominantly with overexpression and/or structural modifications of the chromosomal AmpC cephalosporinase (PDC), leading to increased ceftolozane hydrolysis and reduced susceptibility, with the potential for treatment failure [81,178].

**Cefepime-enmetazobactam** (CFP-ETZ) consists of a fourth-generation cephalosporin and enmetazobactam, which is a BLI structurally related to tazobactam and was developed to expand therapeutic options for infections caused by Enterobacterales producing ESBLs, while reducing reliance on carbapenems [23,179].

In 2024, enmetazobactam, in combination with cefepime, received regulatory approval in both the United States and Europe for the treatment of cUTIs, including pyelonephritis, in adult patients. In the European Union, its approved indications also include HAP, VAP, and bacteremia associated with these infections [23,179,180,181].

Enmetazobactam restores the antibacterial activity of cefepime against ESBL-producing pathogens through inhibition of class A β-lactamases, including CTX-M, TEM, and SHV enzymes. In contrast, it lacks activity against class B MBLs and exhibits limited inhibitory effects on class C and class D β-lactamases. Nevertheless, the clinical activity of the CFP-ETZ combination against organisms producing AmpC and certain OXA enzymes is partially maintained owing to the intrinsic stability of cefepime to hydrolysis by these β-lactamases [179].

In vitro studies have demonstrated substantial activity of the CFP–ETZ combination against OXA-48-producing Enterobacterales, largely owing to the intrinsic stability of cefepime and the inhibition of co-produced ESBLs. In contrast, activity against KPC-producing isolates is more limited, and the combination is generally ineffective against NDM- and VIM-producing organisms. Furthermore, it exhibits limited activity against *P. aeruginosa* and *A. baumannii* (Table 1). As the spectrum of activity is primarily directed against GN pathogens, additional antimicrobial therapy may be required when concomitant infection with GP or anaerobic microorganisms is suspected [179].

Baseline resistance to CFP-ETZ is primarily attributable to the production of β-lactamases that hydrolyze cefepime and are not inhibited by enmetazobactam, particularly MBLs and most KPC variants [182,183]. Reduced outer membrane permeability is another key factor contributing to resistance, and alterations in or even the loss of the OmpK35 and OmpK36 porins significantly reduce the penetration of cefepime into the periplasmic space, particularly in isolates that already produce β-lactamases [182,184]. In the case of some carbapenemase-producing *K. pneumoniae* strains, the status of the porins has been reported to play a greater role than the β-lactamase profile in determining susceptibility. Isolates belonging to sequence types that do not exhibit such porin defects remained more susceptible, despite comparable ESBL profiles [184]. Other important factors include the overexpression of efflux pumps, particularly the AcrAB–TolC system, modifications in PBP3, as well as the overproduction or co-production of other β-lactamases; all of these are associated with reduced susceptibility to this combination [182].

Regarding the in vitro (laboratory-selected) resistance, the tendency for resistance to develop appears to be low, given that in vitro studies have shown that spontaneous resistance occurs at a rate similar to or lower than that observed with cefepime alone. Among the small number of isolates that did become resistant, they did so by developing mutations in the OmpK porins and in the genes encoding the efflux pumps [179].

Clinical evidence regarding the treatment-emergent resistance to CFP-ETZ remains limited. In the Phase III ALLIUM trial, increases in CFP-ETZ MICs during or after therapy were infrequent, but the underlying resistance mechanisms were not investigated [179,182].

Given the increasing use of this agent as a carbapenem-sparing option, in vitro susceptibility testing prior to administration remains warranted, especially in the case of infections caused by carbapenemase-producing organisms [183].

### 4.2. Other New Therapeutic Options

#### 4.2.1. Non-β-Lactam Antibiotics

##### Plazomicin

Plazomicin is a next-generation aminoglycoside that was introduced relatively recently into clinical practice. It received FDA approval in 2018 for the treatment of cUTIs, including pyelonephritis, caused by susceptible Enterobacterales in adult patients with limited or no alternative therapeutic options. However, plazomicin has not been approved by the EMA [84].

Aminoglycosides are a well-established class of antimicrobial agents that were introduced shortly after the advent of β-lactam antibiotics but possess a fundamentally different mechanism of action. These bactericidal agents exert their activity by binding to the 30S ribosomal subunit, thereby disrupting protein synthesis and ultimately leading to bacterial cell death. As with other antimicrobial classes, bacterial resistance to aminoglycosides has evolved through multiple mechanisms, including target-site modifications that impair antibiotic binding, alterations in membrane permeability and/or increased efflux pump activity that limits intracellular drug accumulation, and, most importantly, the production of aminoglycoside-modifying enzymes (AMEs), which enzymatically inactivate the antibiotic before it reaches its ribosomal target [185].

Traditionally, aminoglycosides such as gentamicin, amikacin, and tobramycin have been used primarily in combination with other antimicrobial agents for the treatment of severe infections caused by GP and GN pathogens, including CROs. Their frequent use in combination with β-lactams is supported by the well-established synergistic interaction between these two antibiotic classes. This synergy results principally from the complementary mechanisms of action of β-lactams and aminoglycosides, whereby β-lactam-mediated disruption of bacterial cell wall synthesis enhances cell permeability and facilitates intracellular aminoglycoside uptake, resulting in more effective bacterial killing. Unfortunately, resistance to aminoglycosides is highly prevalent among MDR and CR GNB. In these organisms, resistance is most commonly mediated by AMEs, which are frequently co-expressed with other antimicrobial resistance determinants [83]. Plazomicin was specifically developed to overcome this limitation. Structural modifications of the molecule confer stability against the action of most clinically relevant AMEs, retaining activity in the presence of approximately 15 of the 17 currently recognized enzymes [52,84,185]. In addition, because its mechanism of action is distinct from that of β-lactam antibiotics, plazomicin is unaffected by β-lactamases, including carbapenemases, the predominant antibiotic resistance mechanisms among Enterobacterales, which may compromise the activity of many β-lactam-based therapeutic agents, including several recently introduced BL/BLI combinations. Consequently, it represents a valuable therapeutic option for infections caused by ESBL-producing and CRE, including KPC-producing isolates and strains resistant to other aminoglycosides [83]. A notable exception is represented by NDM-producing organisms, as genes encoding NDM MBLs are frequently co-located with 16S rRNA methyltransferases, which confer high-level resistance to virtually all clinically available aminoglycosides, including plazomicin [63,186] (Table 1). Furthermore, plazomicin may be affected by non-specific efflux mechanisms, which are frequently encountered in NF-GNB, particularly *P. aeruginosa* and *A. baumannii*. As a result, its activity against these pathogens is limited, and it is generally not considered a preferred therapeutic option for infections caused by these organisms [185].

The pharmacokinetic properties of plazomicin also influence its clinical utility. As with other aminoglycosides, owing to its limited tissue penetration, with the exception of the renal cortex where high concentrations are achieved, its distribution into extrarenal tissues is relatively restricted. Consequently, its principal clinical application remains the treatment of UTIs [185]. However, unlike earlier aminoglycosides, which are generally administered as part of combination regimens, the favorable spectrum of activity and enhanced stability against AMEs allow plazomicin to be used as targeted monotherapy in selected cases. In particular, it represents a valuable therapeutic option for cUTIs caused by MDR or XDR Enterobacterales when alternative treatment options are limited [63,84].

Although plazomicin retains activity against most CRE, reduced susceptibility has been reported, particularly among isolates harboring 16S rRNA methyltransferases. These enzymes represent the primary mechanism of clinically relevant resistance to plazomicin by modifying the aminoglycoside binding site on the bacterial ribosome. In addition, other mechanisms may contribute to reduced plazomicin activity, including the production of rare AMEs capable of inactivating plazomicin. As previously mentioned, resistance has also been observed more frequently among NDM-producing isolates, largely due to the frequent co-carriage of genes encoding 16S rRNA methyltransferases rather than a direct effect of carbapenemase production [187].

##### Eravacycline

Eravacycline is a fully synthetic fluorocycline antibiotic belonging to the tetracycline class and is structurally related to tigecycline. However, targeted modifications of its tetracycline core confer improved intrinsic antibacterial potency and enhanced stability against several mechanisms responsible for tetracycline resistance [188,189,190].

Tetracyclines are a long-established class of broad-spectrum antimicrobial agents with activity against a wide range of GP and GN bacteria [191]. Their antibacterial effect relies primarily on inhibition of protein synthesis through reversible binding to the bacterial 30S ribosomal subunit, although additional interaction with the 50S subunit has also been suggested. This binding interferes with aminoacyl-tRNA accommodation and prevents peptide chain elongation, ultimately impairing bacterial protein synthesis [187].

Before the development of eravacycline and the introduction of novel BL/BLIs, older tetracycline derivatives, particularly tigecycline and minocycline, represented valuable therapeutic options for infections caused by MDR and CROs, including CRAB. These agents were frequently incorporated into combination regimens for the management of DTR infections. However, their role has progressively diminished, especially in CRE infections, following the availability of more effective targeted therapies [84]. In addition, important pharmacological limitations, including their predominantly bacteriostatic activity and reduced serum concentrations resulting from extensive tissue distribution—particularly observed with tigecycline—have limited their utility in severe systemic infections such as bacteraemia and sepsis. Nevertheless, these tetracycline derivatives remain clinically relevant in specific settings where newer BL/BLI agents provide limited benefit, particularly for infections caused by CRAB, usually in combination with at least one other antimicrobial agent.

Resistance to tetracyclines is mediated through several mechanisms, most commonly encoded by genes acquired via mobile genetic elements such as plasmids and transposons. The principal mechanisms include active drug efflux, which represents the predominant pathway of resistance, ribosomal target protection or modification, and enzymatic degradation through tetracycline-inactivating destructase enzymes [185,187]. Eravacycline was specifically engineered to overcome the major resistance determinants limiting the efficacy of previous tetracycline agents [188]. Its structural modifications preserve activity against organisms expressing ribosomal protection proteins and most tetracycline-specific efflux mechanisms, particularly non-RND efflux pumps [190]. Importantly, eravacycline activity is not affected by carbapenemase production, supporting its potential role against carbapenemase-producing carbapenem-resistant pathogens, especially CRE [69].

Eravacycline exhibits broad-spectrum antimicrobial activity against GN, GP and anaerobic bacteria, including MDR isolates; its in vitro efficacy has been demonstrated against CRE, including KPC-, OXA- and some MBL-producing strains, as well as against *A. baumannii* [84,188,192] (Table 1). However, consistent with the intrinsic limitations of the tetracycline class, eravacycline retains limited activity against *Proteeae* and lacks clinically relevant activity against *P. aeruginosa* isolates.

Eravacycline was approved by the FDA in 2018 for the treatment of cIAIs, with subsequent marketing authorization granted in the United Kingdom and the European Union. Similar to other tetracyclines, although to a lesser extent, eravacycline exhibits a large volume of distribution and, following administration, is rapidly distributed into various tissues, where it achieves high concentrations; however, plasma and urinary concentrations remain relatively low. This pharmacokinetic/pharmacodynamic (PK/PD) profile likely makes eravacycline an inappropriate choice for the treatment of sepsis or UTIs [189]. Indeed, eravacycline failed to obtain approval for the treatment of cUTIs, as non-inferiority compared with fluoroquinolone- and carbapenem-class comparator agents could not be demonstrated for this indication [63,69].

Moreover, although in vitro and in vivo studies have demonstrated improved activity against CRAB and CRE, higher concentrations in lung tissue, and fewer adverse effects with eravacycline compared with tigecycline, clinical data remain limited, and further studies are required to confirm the clinical efficacy of eravacycline, particularly in CRAB infections [193].

Currently, the use of eravacycline for the treatment of infections other than cIAIs, including HAP/VAP, skin and soft tissue infections (SSTIs), and infections caused by MDR *A. baumannii*, is considered off-label. In these clinical scenarios, eravacycline should be used with caution, only when other therapeutic options are unavailable or unsuitable, and generally as part of a combination antimicrobial regimen. In keeping with this, the American guidelines for the treatment of infections caused by MDR organisms suggest that eravacycline may represent a potential therapeutic option for infections caused by CRE, with the exception of UTIs and BSIs; however, this recommendation is not included in the European guidance [84].

Although eravacycline was developed to overcome several resistance mechanisms affecting earlier tetracyclines, clinically relevant resistance has been described among both Enterobacterales and *A. baumannii*. Reduced susceptibility is primarily mediated by overexpression of multidrug efflux systems, including AcrAB-TolC in *K. pneumoniae* and AdeABC in *A. baumannii*, as well as by enzymatic inactivation mediated by plasmid-encoded *tet(X)* variants [52,187]. While these mechanisms have been confirmed in clinical isolates, the emergence of resistance during eravacycline therapy remains insufficiently characterized due to limited clinical experience.

#### 4.2.2. New β-Lactam Antibiotics

##### Cefiderocol

Although the introduction of novel BL/BLI combinations has considerably expanded the therapeutic options for infections caused by MDR-GNB, particularly CR pathogens, important therapeutic gaps remain. These limitations are mostly relevant for infections caused by MBL-producing organisms and CRAB, for which the number of reliably active agents remains comparatively limited [194].

Cefiderocol, a novel siderophore cephalosporin, was specifically developed to address these unmet clinical needs. Its unique mechanism of cell entry, together with its enhanced stability against hydrolysis by a broad range of both serine- and metallo-β-lactamases, confers broad in vitro activity against MDR GNB, including CRE, *P. aeruginosa* and *A. baumannii* [50,195,196]. Indeed, cefiderocol exhibits one of the broadest in vitro spectra among currently approved agents against CR-GNB (Table 1). However, this broad microbiological activity does not uniformly translate into comparable clinical benefit across pathogens and clinical settings, with particularly heterogenous outcome data reported for infections caused by *A. baumannii* [197,198,199]. More recently, the GAME CHANGER randomized trial demonstrated non-inferiority of cefiderocol to standard-of-care therapy for 14-day mortality in hospital- or healthcare-associated GN BSI, but did not demonstrate superiority, including in the subgroup with carbapenem-resistant organisms [200].

This therapeutic agent received approval from both the FDA and the EMA [194,201]. The FDA initially approved cefiderocol in 2019 for the treatment of cUTIs, including pyelonephritis, with its indication subsequently expanded in September 2020 to include HAP and VAP [202]. In the European Union, cefiderocol is approved for the treatment of infections caused by aerobic GN organisms in adults with limited treatment options [201].

Structurally, cefiderocol shares similarities with cefepime and ceftazidime but incorporates several strategic modifications that distinguish it from earlier-generation cephalosporins. These structural adaptations confer enhanced stability against hydrolysis by numerous serine- and metallo-β-lactamases, including clinically important carbapenemases such as OXA, KPC, NDM, and VIM [194,196]. However, this stability against β-lactamases is not absolute, as increased cefiderocol MICs have been reported in some isolates, particularly those producing NDM enzymes, but also in association with certain other β-lactamases or their variants.

In addition, the incorporation of a catechol siderophore moiety enables cefiderocol to chelate ferric iron, allowing the resulting complex to be actively transported across the outer membrane via bacterial iron uptake systems. This siderophore-mediated uptake, commonly referred to as a “Trojan horse” mechanism, differs from that of conventional β-lactams, which rely primarily on passive diffusion through porin channels for cell entry. Consequently, cefiderocol uptake may be less affected by permeability defects associated with porin loss or alterations [194,203]. Once within the periplasmic space, iron dissociates from the complex, permitting the cephalosporin moiety to bind predominantly to PBP3, thereby inhibiting peptidoglycan synthesis and disrupting bacterial cell-wall formation. Together with its enhanced β-lactamase stability, this active uptake mechanism makes cefiderocol less affected than conventional β-lactams by several resistance mechanisms, including reduced outer-membrane permeability and increased efflux activity. Nevertheless, these mechanisms are not completely circumvented and, particularly when combined with alterations in siderophore-mediated iron uptake, certain β-lactamase production, or target modifications, may contribute to reduced cefiderocol susceptibility [202,203,204].

In vitro surveillance studies have demonstrated that cefiderocol possesses broad-spectrum activity against a wide range of clinically relevant GN pathogens, including members of the Enterobacterales order—such as *E. coli*, *Klebsiella* spp., *Citrobacter* spp., *Enterobacter* spp., *Serratia* spp., *Morganella morganii*, and *Proteus* spp.—as well as several clinically important NF-GNB, including isolates exhibiting MDR and DTR resistance phenotypes, such as *P. aeruginosa*, CRAB and *Stenotrophomonas maltophilia* [194]. However, susceptibility is not universal and varies according to species, carbapenemase type, and associated resistance mechanisms.

Most recent clinical guideline recommendations regarding cefiderocol therapy therefore differ by pathogen and resistance mechanism. According to the 2026 Infectious Disease Society of America (IDSA) guidance, for invasive infections caused by NDM-producing Enterobacterales, cefiderocol is one of the recommended therapeutic options (alongside AZT-AVI). For infections caused by DTR *P. aeruginosa*, the recommendation for cefiderocol therapy depends on the site of infection and the mechanism of resistance. It is among the preferred options for cUTI, whereas in the case of infections outside the urinary tract, it is considered an alternative when treatment with newer antipseudomonal β-lactams is not feasible. Nevertheless, in infections caused by MBL-producing *P. aeruginosa*, for which most other currently available newer antipseudomonal β-lactams lack activity, cefiderocol is the preferred therapeutic option [73].

The role of cefiderocol in the management of CRAB infections remains less clearly defined. In the CREDIBLE-CR trial, a numerical excess in all-cause mortality was observed in the cefiderocol group compared with best available therapy, with the imbalance occurring predominantly among patients with *Acinetobacter* infections. However, the *Acinetobacter* subgroup was relatively small; the trial was descriptive rather than powered to establish organism-specific superiority or inferiority, and comparator regimens were heterogeneous [197]. Subsequent observational studies and meta-analyses have reported more favorable outcomes with cefiderocol-based therapy in some CRAB populations, but these data are limited by their predominantly observational design, heterogeneity in patient characteristics, infection sites, disease severity, concomitant therapy, and comparator regimens. [199,202,205]. Thus, the available evidence remains inconclusive. Current IDSA guidance positions cefiderocol, generally in combination with another active agent, as an alternative for invasive CRAB infections when SUL-DUR cannot be used because of resistance or as interim therapy while access to SUL-DUR is pending [73]. Additional comparative clinical studies are required to better define its optimal role in severe CRAB infections.

Despite its broad antimicrobial spectrum and enhanced stability against multiple resistance determinants, resistance to cefiderocol has increasingly been reported. Several mechanisms have been implicated in reduced susceptibility, including the production of specific β-lactamases, such as certain variants of KPC, AmpC, SHV/PER ESBLs, NDM, and OXA-427, as well as mutations affecting the expression or function of siderophore receptors involved in antibiotic uptake. Additional mechanisms—including alterations in porin expression, efflux pump overactivity, and modifications of PBP3—may also contribute to reduced susceptibility, although these mechanisms alone are generally insufficient to increase the MIC beyond clinically relevant PK/PD targets [203].

The contribution of β-lactamases to cefiderocol resistance is further supported by in vitro experimental studies demonstrating restoration of antimicrobial activity following the addition of BLIs. Specifically, avibactam enhances cefiderocol activity against isolates producing SBLs, whereas dipicolinic acid potentiates its activity against MBL-producing isolates [203]. These findings demonstrate that cefiderocol is not completely stable to all β-lactamases and support a contributory role of β-lactamase-mediated hydrolysis in the development of cefiderocol resistance.

Furthermore, cefiderocol heteroresistance has also been increasingly recognized, particularly among CR isolates and most notably CRAB, whereas considerably lower rates have been observed in isolates susceptible to both carbapenems and expanded-spectrum cephalosporins. Systematic investigations have demonstrated the coexistence of cefiderocol-susceptible and cefiderocol-resistant subpopulations within isolates that may be categorized as susceptible by conventional susceptibility testing [203]. More recent clinical investigations have similarly identified heteroresistance among CRAB isolates and have raised the possibility that it may contribute to treatment failure [206]. However, its prevalence varies substantially according to the organism, study population, and methodology used for detection, and its independent effect on clinical outcomes has not yet been established. Accordingly, the clinical significance of cefiderocol heteroresistance remains uncertain and warrants further investigation.

## 5. Laboratory Detection of Carbapenemase-Producing GNB

Given the threat posed by CROs to both individual patient health and public health, as the infections caused by these organisms are associated with increased morbidity and mortality [1,2], clinical laboratories can play an essential role in limiting their dissemination and improving patient outcomes. This may be achieved through the early and accurate detection of such pathogens and the differentiation of carbapenemase-producing organisms (CP-CROs) from non-carbapenemase-producing ones (non-CP CROs) [207,208].

On the one hand, in most CP-CRO strains, carbapenemase genes are commonly located on MGEs, which facilitate the horizontal transfer of resistance determinants among bacterial strains belonging to the same or different species, and even genera. This contributes significantly to the spread of CROs among patients [207,208]. Consequently, rapid and accurate identification of these organisms is essential, as it necessitates the prompt implementation of enhanced infection prevention and control (IPC) measures, including the early initiation of contact precautions and the cohorting of both patients and the nursing staff involved in their care. Furthermore, by identifying CP-CROs and characterizing carbapenemase types, the clinical laboratory enables surveillance of CRO prevalence within a healthcare facility and supports the early detection of emerging carbapenemase variants that may arise over time [208].

On the other hand, given that the limited therapeutic options introduced in recent years—primarily consisting of combinations of older β-lactam antibiotics with novel BLIs (such as ceftazidime- or aztreonam/avibactam, meropenem/vaborbactam, and imipenem/relebactam)—are not universally active against all CP-CROs regardless of carbapenemase type, but instead exhibit selective activity (Table 1), the rapid identification of the specific carbapenemase produced may substantially improve clinical management and consequently the patients outcome in infections caused by these organisms [209].

Although the carbapenemase type may sometimes be inferred from routine antimicrobial susceptibility testing (AST), this approach is not always reliable. Many strains produce multiple carbapenemases or co-produce a carbapenemase alongside other β-lactamases, such as ESBLs or AmpC enzymes, which may lead to misleading phenotypic profiles [209]. Moreover, AST results typically require at least 18–24 h to obtain. Early identification of the carbapenemase type can therefore guide empirical therapy decisions, particularly in determining whether one of the newer therapeutic options may be appropriate prior to the availability of definitive AST results [208].

In this context, the clinical laboratory plays a critical role not only in epidemiological surveillance and IPC efforts, but especially in antimicrobial stewardship initiatives. These efforts aim to ensure the judicious use of novel therapeutic agents and to prolong their clinical utility by preventing the emergence of resistance, which has already been reported [81].

The identification of CP-CROs can be achieved either through currently available phenotypic methods, which assess the behavior of microorganisms in the presence of carbapenems (some of these tests have been in use for over a decade), or through genotypic approaches, which directly detect the genes responsible for carbapenemase production in the respective strains.

### 5.1. Phenotypic Methods

The phenotypic assays vary based on the method they use in order to determine the presence of a carbapenemase in the tested strain. There are assays that are growth based, which are measuring the carbapenem resistance by assessing the bacterial growth in the presence of a carbapenem, like modified Hodge test (MHT), combined disc test (CDT) and modified carbapenem inactivation method (mCIM); assays based on hydrolysis exerted by the enzyme if present, which determine the presence of the carbapenem-hydrolysis products, like the colorimetric carba NP assay and proteomic-based MALDI-TOF MS and the immunochromatographic assays that use specific antibodies in order to identify the specific carbapenemases, the lateral flow immunoassays (LFIA). In the case of some of the growth-based (MHT, CIM/mCIM) and hydrolysis-based (carbaNP) assays, their original formulations were not capable of differentiating between carbapenemases, or even between the major classes of these enzymes. As this distinction has become increasingly important—due to the rising prevalence of diverse carbapenemase types in certain regions and the emergence of novel BL/BLI combinations—these traditional methods have undergone numerous modifications. Such adaptations have been introduced either to improve test performance with respect to specific bacterial species or to enable the differentiation of carbapenemase classes, typically through the incorporation of specific enzyme inhibitors [207,208].

Although the majority of these tests are relatively easy to perform, requiring minimal expertise and resources that are generally available in most laboratories, and demonstrate high sensitivity and specificity—dependent on the bacterial species and the type of carbapenemase produced—each method has its own advantages and limitations which must be carefully considered when a laboratory seeks to implement such tests in routine practice (Table 3).

### 5.2. Genotypic Methods

Genotypic methods are primarily based on nucleic acid amplification technologies, most notably polymerase chain reaction (PCR), performed either as an in-house developed multiplex real-time PCR assay or as syndromic assays using one of the commercially available platforms, which combine PCR with DNA hybridisation techniques. The presence of carbapenemase-encoding resistance genes may also be identified through whole-genome sequencing (WGS), a next-generation sequencing (NGS)-based approach that enables the analysis of the entire bacterial genome [62].

Although these approaches offer greater analytical precision and considerably shorter TAT compared to traditional phenotypic assays, most of them having the major advantage of being used directly on patient samples, their application for the identification of CP-CROs in clinical practice remains a subject of debate. This is due to the fact that the detection of a resistance gene does not necessarily translate into phenotypic resistance, as the gene may be either unexpressed or expressed at low levels [209]. Consequently, a paradoxical, undesired effect may occur, namely an increased use of the last-resort antibiotics.

A further major limitation of these approaches is their substantially greater cost relative to phenotypic assays, both in terms of per-test reagent expenses and the overall investment required for specialized equipment. In addition, these methods demand considerable technical expertise for both assay performance and result interpretation, particularly in the case of laboratory-developed PCR assays and WGS [208].

Another important constraint is the restricted spectrum of detectable carbapenemases, which is inherently determined by primer selection for the in-house PCR-based assays, requiring advance knowledge of the specific targets, or by the predefined target panels incorporated into commercial syndromic platforms. These panels are generally designed to identify the most prevalent carbapenemase genes, typically including KPC, NDM, IMP, VIM and OXA-type enzymes, and are unable to detect genes encoding rare, unusual carbapenemases or new variants [208].

### 5.3. Selecting the Most Appropriate Test

There is currently no standardized approach to the detection and characterization of CP-CROs, as each laboratory may select the test, or combination of tests, that is most appropriate within its specific operational context [81]. This variability arises, on the one hand, from the fact that each method presents distinct advantages and limitations, some of which are directly related to test performance in relation to specific microorganisms or particular types of carbapenemases. On the other hand, it is well established that the epidemiology of CP-CROs is highly heterogeneous across different regions of the world; consequently, diagnostic strategies that are suitable in certain settings may not be optimal in others. Nevertheless, several key considerations should be taken into account.

From an epidemiological perspective, it is important to recognize that in certain regions there is a predominance of strains producing multiple carbapenemases or exhibiting carbapenem resistance as a result of the combined effects of carbapenemase production and additional resistance mechanisms, such as porin loss or the overexpression of efflux pumps [81]. In such contexts, diagnostic strategies may differ substantially from those employed in regions where these phenomena are infrequent. For example, laboratories operating in these settings may opt for LFIA, which enable the simultaneous detection of the five most commonly encountered carbapenemases, or for PCR-based methods, which can also identify the presence of additional resistance mechanisms.

Furthermore, the choice of diagnostic method may also vary depending on the clinical context in which a CRO is isolated. For instance, in patients presenting with bacteremia or sepsis, when blood cultures yield CROs, the time to initiation of the most appropriate antimicrobial therapy is critical. Early administration of effective therapy has been shown to significantly improve survival outcomes in these patients [212,213]. Therefore, in such scenarios, the selected diagnostic method should have a minimal turnaround time (TAT), typically favoring rapid phenotypic assays that provide results within minutes to hours (as opposed to those requiring bacterial growth), or alternatively, genotypic methods. Moreover, among the available techniques, genotypic and immunochromatographic methods offer the additional advantage of being applicable directly to clinical specimens, without the need for prior bacterial culture, an aspect of particular relevance in regions with a high burden of CROs.

Finally, the economic context must also be taken into account when selecting an appropriate diagnostic approach. For certain assays—particularly genotypic methods—the cost per test can be considerably higher, rendering them impractical for implementation in laboratories operating in resource-limited or low-income settings. In such circumstances, the use of multiple phenotypic tests in parallel may be considered a viable alternative, as highlighted in studies conducted in low-resource settings [211].

## 6. Discussion

CR-GNB remain a major clinical challenge, particularly in HAIs, where antimicrobial selective pressure and opportunities for transmission favor the emergence and dissemination of MDR pathogens. Although the introduction of newer antimicrobial agents and BL/BLI combinations has substantially expanded therapeutic options, most represent extensions of existing antimicrobial agents or classes rather than entirely new classes of antibiotics. Their activity remains strongly dependent on the bacterial species, carbapenemase type, and coexisting resistance mechanisms, including altered permeability, the activity of the efflux pumps, target site modifications, and β-lactamase expression. Consequently, the CR phenotype alone is insufficient to guide selection of a newer antimicrobial agent.

Therapeutic options have improved particularly for KPC- and OXA-48-like-producing Enterobacterales, whereas important gaps remain for NDM-producing ABC and MBL-producing *P. aeruginosa*. The interpretation of resistance to newer agents should also distinguish baseline, laboratory-selected, and treatment-emergent resistance, as these categories provide different information regarding empirical reliability, potential resistance pathways, and therapeutic durability. Treatment-emergent resistance is documented for some agents, whereas the limited clinical experience with more recently introduced therapies should not be interpreted as evidence of a low risk of resistance.

Rapid carbapenemase identification may support earlier therapeutic decisions, but it cannot replace agent-specific AST, because susceptibility may be modified by additional resistance determinants. AST therefore remains essential for definitive treatment selection and should be repeated when resistance emergence is suspected. Similar caution is required when interpreting cefiderocol activity. Although it retains broad in vitro activity against many CR-GNB, its optimal role in CRAB infections and the clinical significance of heteroresistance remain incompletely defined.

Local epidemiological surveillance is equally important because the distribution of carbapenemases and associated resistance mechanisms varies across institutions and geographical regions. Integration of resistance-mechanism characterization, AST, surveillance, antimicrobial stewardship, and infection prevention is therefore necessary to guide appropriate therapy and preserve the activity of available agents.

Several limitations should be considered when interpreting this narrative review. Reliance primarily on PubMed and English-language full-length publications may have limited literature coverage despite supplementary reference screening. In addition, heterogeneity in study design, pathogens, resistance mechanisms, clinical settings, and susceptibility-testing methods limits direct comparisons between agents. Clinical data remain limited for several recently introduced antimicrobials, while some resistance mechanisms have been described predominantly in vitro or in small isolate collections. Accordingly, the comparative synthesis should be interpreted in relation to the type and strength of evidence available for each agent.

Future studies should prioritize therapeutic strategies for NDM-producing ABC and MBL-producing *P. aeruginosa*, prospective studies stratified by resistance mechanism, and analysis of paired pre- and post-treatment isolates. Further investigation is also required to clarify the clinical significance of cefiderocol heteroresistance, evaluate the potential role of CFP-ZID and eravacycline in CRAB infections, and determine whether locally adapted diagnostic strategies improve treatment selection and patient outcomes.

## 7. Conclusions

Carbapenem resistance in GNB should be interpreted according to the bacterial species, carbapenemase type, and accompanying resistance mechanisms rather than as a uniform therapeutic phenotype. Although newer antimicrobials have substantially expanded treatment options, important therapeutic gaps persist and resistance may be present before clinical use or emerge during therapy. Mechanism-directed diagnostics should therefore complement, but not replace, agent-specific AST. Sustainable management of CR-GNB infections will require the coordinated use of rapid microbiological diagnostics, continuous epidemiological surveillance, antimicrobial stewardship, and rigorous IPC. These measures remain essential for optimizing targeted therapy and preserving the effectiveness of both existing and future antimicrobial agents.

## Figures and Tables

**Figure 1:**
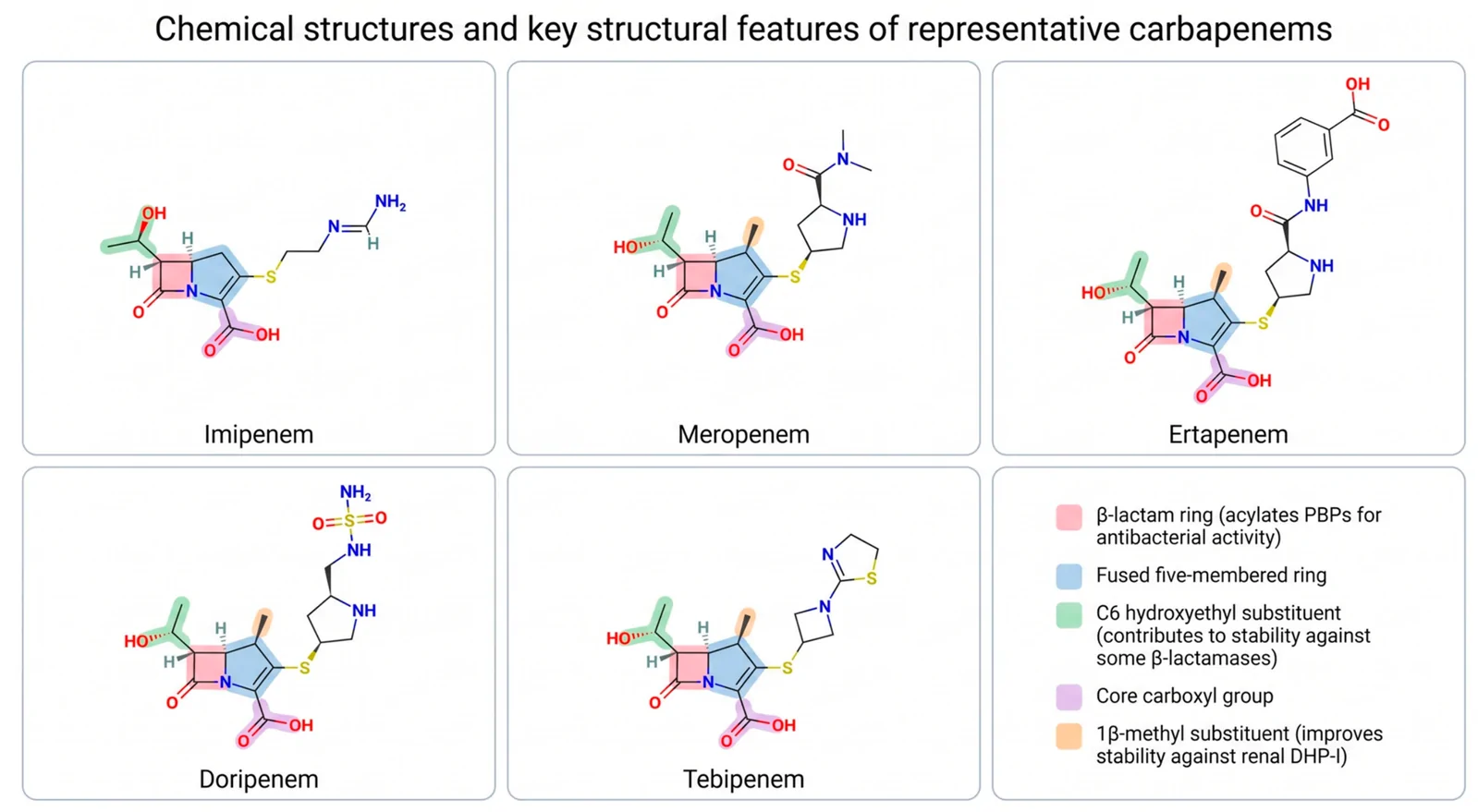
Chemical structures and key structural features of representative carbapenems: imipenem, meropenem, ertapenem, doripenem and tebipenem (the active form of the oral prodrug tebipenem pivoxil). Coloured highlights indicate the β-lactam ring, which acylates penicillin-binding proteins and is responsible for antibacterial activity (pink); the fused five-membered pyrroline ring (blue); the C6 hydroxyethyl substituent, trans to H5, which contributes to β-lactamase stability (green); the carboxyl group of the bicyclic core (purple); and the 1β-methyl substituent, which confers stability against renal dehydropeptidase-I (DHP-I) and is absent in imipenem, explaining its co-administration with cilastatin (orange). The C2 side chains modulate antibacterial spectrum, pharmacokinetics and stability. Structures were drawn based on PubChem 2D depictions (CIDs 104838, 441130, 150610, 73303 and 9800194). Created in BioRender. Vulpie, S. (2026) https://BioRender.com/zeru7i3.

**Figure 2:**
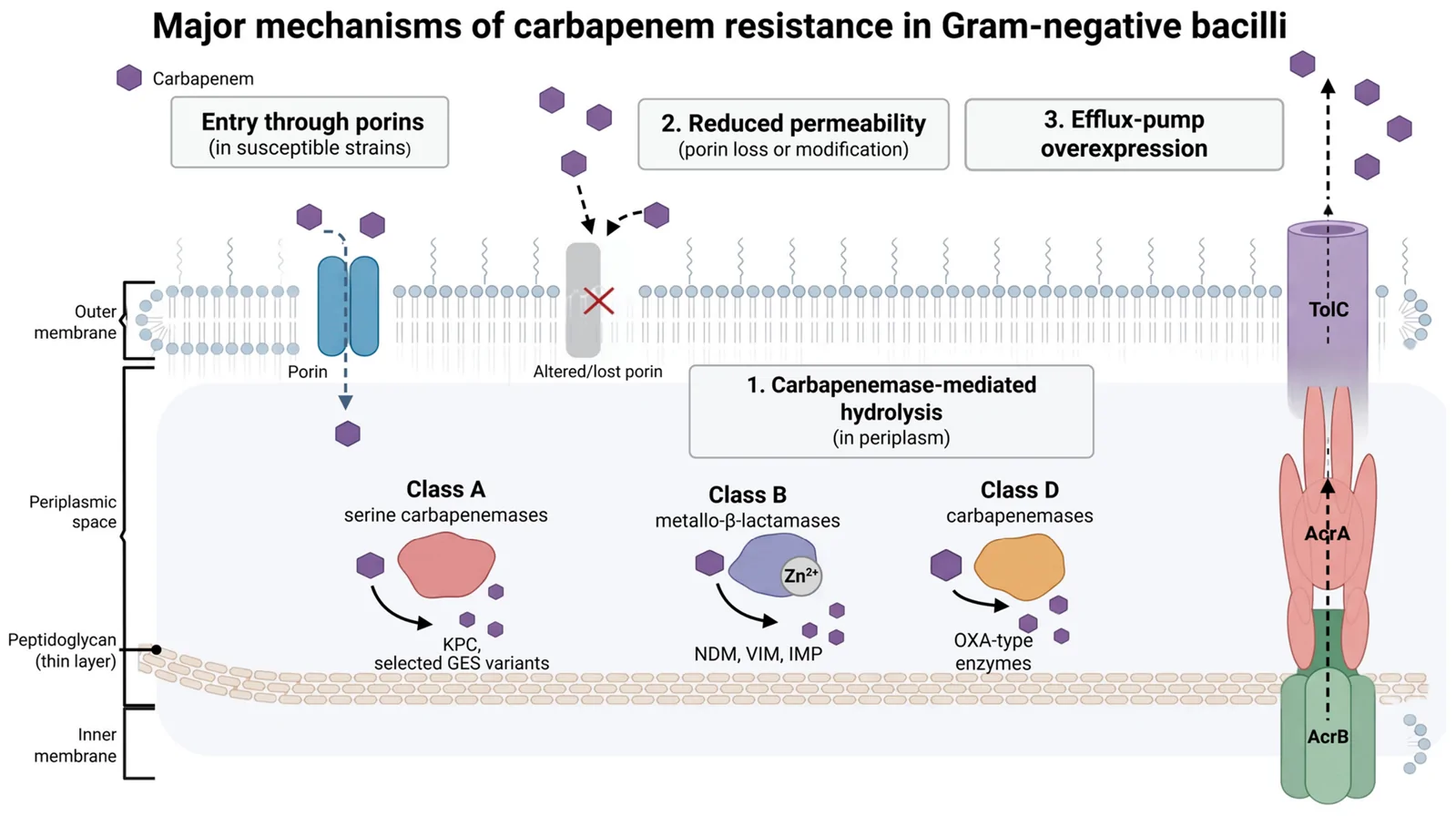
Major mechanisms of carbapenem resistance in Gram-negative bacilli. In susceptible strains, carbapenems cross the outer membrane mainly through porin channels. The principal mechanism addressed in this review is (1) carbapenemase-mediated hydrolysis in the periplasmic space by class A serine carbapenemases (e.g., KPC and selected GES variants), class B metallo-β-lactamases (e.g., NDM, VIM and IMP; Zn^2+^-dependent) and class D carbapenemases (OXA-type enzymes, e.g., OXA-48-like and OXA-23). Additional mechanisms include (2) reduced outer-membrane permeability due to porin loss or modification (e.g., OprD in *P. aeruginosa*, OmpK35/OmpK36 in *K. pneumoniae*) and (3) overexpression of efflux pumps (e.g., AcrAB–TolC), which frequently act together with other β-lactamases such as AmpC or ESBLs. Created in BioRender. Vulpie, S. (2026) https://BioRender.com/rnf1kwy.

**Table 1:** Comparative activity, approved indications and main limitations of the antimicrobial agents available for CR-GNB infections.

Antibacterial Agent	KPC	MBL (NDM/VIM/IMP)	OXA-48-Like	DTR-PA	CRAB	Approved Indications	Clinical Limitations
CAZ-AVI	**+**	**-**	**+**	**±**	**-**	• cUTI, cIAI (+MTZ) (FDA 2015; EMA 2016) • HAP/VAP (2018)	• not active against MBLs or CRAB • best documented treatment-emergent resistance and cross-resistance-requires repeat AST during treatment
ATM-AVI	**+**	**+**	**+**	**-**	**-**	• cIAI, cUTI incl. pyelonephritis, HAP/VAP, limited-option GN infections (EMA 2024) • cIAI (+MTZ) (FDA 2025)	• not active against *Acinetobacter* spp. • poor activity against *P. aeruginosa*, especially MBL-producing strains (usually other carbapenem resistance mechanisms associated) • regulatory indications differ by region
IMI-REL	**+**	**-**	**-**	**±**	**-**	• cUTI, cIAI (FDA 2019); HAP/VAP (2020); EMA 2020 (incl. BSI and other limited-option GN infections)	• not active against Morganellaceae, CRAB, OXA-48-like and MBLs • not active against *P. aeruginosa* producing certain class A and D carbapenemases (VEB, GES, OXA-type) or MBLs
SUL-DUR	**-**	**-**	**-**	**-**	**+**	• HAP/VAP due to ABC/CRAB (FDA 2023) • not authorized by EMA	• only for ABC infections • not active against MBLs (incl. NDM-producing ABC strains) • access differs by region
CFP-ZID	**+**	**+** **	**+**	**+**	**±**	• cUTI incl. pyelonephritis (FDA 2026) • not authorized by EMA	• currently indication limited only to cUTI • access differs by region
MEM-VAB	**+**	**-**	**-**	**-**	**-**	• cUTI (FDA 2017) • cUTI incl. pyelonephritis, cIAI, HAP/VAP, BSI and other limited-option GN infections (EMA 2018)	• activity mainly against KPC-producing Enterobacterales • not active against OXA-48-like, MBLs, DTR-PA or CRAB
CTL-TZB	**-**	**-**	**-**	**+** *	**-**	• cIAI, cUTI (FDA 2014; EMA) • HAP/VAP (2019)	• antipseudomonal agent • no carbapenemase -directed activity • possible PDC mutations or overexpression during therapy, repeat AST required
CFP-ETZ	**-**	**-**	**±**	**-**	**-**	• cUTI incl. pyelonephritis (FDA/EMA 2024) • HAP/VAP, BSI (EU)	• primarily an ESBL-directed carbapenem-sparing agent • AST before using in CPE infections
CFD	**+**	**+**	**+**	**+**	**±**	• cUTI (FDA 2019); HAP/VAP (2020); EMA 2020	• mixed outcomes in CRAB infection warrant further clinical studies to define the optimal role of CFD in severe disease • heteroresistance identified, predominantly among CR isolates
PLZ	**+**	**-** ***	**±**	**-**	**-**	• cUTI incl. pyelonephritis, in patients with limited or no other therapeutic options (FDA 2018) • not authorized by EMA	• indication limited only to cUTI caused by Enterobacterales • access differs by region
ERV	**+**	**+**	**+**	**-**	**±**	• cIAI (FDA 2018; EMA/UK)	• low urinary and plasma concentrations • off-label use outside cIAI

The information presented in this table was obtained from the sources cited in the main text within the respective sections addressing each therapeutic option. Abbreviations: KPC–*Klebsiella pneumoniae* carbapenemase; MBL–metallo-β-lactamase; NDM–New Delhi metallo-β-lactamase; VIM–Verona integron-encoded metallo-β-lactamase; IMP-imipenemase; OXA–oxacillinase; DTR-PA–difficult-to-treat *Pseudomonas aeruginosa*; CRAB-carbapenem-resistant *Acinetobacter baumannii*; ABC–*Acinetobacter baumannii-calcoaceticus* complex; CPE–carbapenemase-producing Enterobacterales; CAZ-AVI–ceftazidime/avibactam; ATM-AVI–aztreonam/avibactam; CFP-ZID–cefepime/zidebactam; MEM-VAB–meropenem/vaborbactam; IMI-REL–imipenem-cilastatin/relebactam; SUL-DUR-sulbactam–durlobactam; CTL-TZB–ceftolozane/tazobactam; CFP-ETZ–cefepime/enmetazobactam; CFD–cefiderocol; PLZ–plazomicin; ERV–eravacycline; MTZ–metronidazole; FDA-U.S. Food and Drug Administration; EMA-European Medicines Agency; EU–European Union; UK–United Kingdom; cUTI–complicated urinary tract infections; cIAI–complicated intra-abdominal infections; HAP/VAP–hospital-acquired pneumonia/ventilator-associated pneumonia; BSI–bloodstream infection; AST–antimicrobial susceptibility testing; +, clinically relevant activity; -, inactive or limited, unreliable activity; ±, variable activity, depending on the isolate, enzyme variant or other underlying resistance mechanisms involved; *, active mainly when non-carbapenemase mechanisms predominate; **, no direct MBL inhibition, activity attributable to the BLE effect of zidebactam; ***, not active against NDM producers, as *bla*NDM is frequently co-carried with 16S rRNA methyltransferase genes.

**Table 2:** Drug, organism, resistance mechanisms, level of evidence and clinical consequences for the therapeutic agents introduced since 2014.

Agent	Organism	Major Resistance Mechanisms	Level of Evidence	Clinical Consequence
CAZ-AVI	KPC-producing *K. pneumoniae*	KPC-2/KPC-3 variants; increased *bla*KPC copy number/expression; OmpK35/OmpK36 alterations; efflux; SHV, CTX-M, VEB, AmpC	In vitro Treatment-emergent	Repeat AST during therapy; “see-saw” effect (transient carbapenem susceptibility, ESBL-like phenotype); false-negative immunochromatographic tests
	*P. aeruginosa*	PDC variants	In vitro Treatment-emergent	Unreliable empirically where PDC variants circulate
ATM-AVI	NDM-producing *E. coli*	PBP3 insertions; CMY-42, CMY-145; CTX-M-15; OmpC/OmpF alterations; efflux	Baseline (described before marketing) Treatment-emergent	Possible cross-resistance to cefepime and cefiderocol (PBP3)
	*K. pneumoniae*	KPC overproduction/variants; CMY-type AmpC; OmpK35/OmpK36 alterations; efflux	Baseline In vitro In vivo (after cefiderocol)	Multifactorial, cumulative resistance
IMI-REL	*K. pneumoniae* and other CR-NME	OXA-48-like; MBLs; SME; OmpK36 ± OmpK35 disruption with KPC (increased copy number, modifications)	Baseline In vitro Clinically evolved	In vitro activity vs. CAZ–AVI-resistant KPC-3 variants
	*P. aeruginosa*	VEB; GES; MBLs (VIM, IMP, NDM); OXA-type carbapenemases	Baseline	Active despite OprD loss/efflux; inactive vs. VEB, GES, MBL, OXA producers; in vitro activity vs. CAZ–AVI/ CTL–TZB-resistant PDC variants
SUL-DUR	*A. baumannii–calcoaceticus* complex	NDM; PBP3 alterations; β-lactamase overproduction; efflux (*adeJ*)	Baseline In vitro Treatment-emergent exceptionally rare	NDM-producing ABC uniformly resistant; PBP3 changes may pre-date exposure
CFP-ZID	Enterobacterales	PBP2 alterations; KPC 270-loop variants; NDM-5/NDM-14 with porin or PBP alterations	In vitro	Unaffected by PBP3 alterations; resistance usually multifactorial
	*P. aeruginosa*	Efflux; PBP2/PBP3 alterations; NDM	In vitro Treatment-emergent	Several determinants needed to abolish activity
MEM-VAB	*K. pneumoniae*	OmpK35/OmpK36 alterations; OmpK36 deletions/duplications	Baseline	OmpK36 alterations compromise clinical effectiveness
CTL-TZB	*P. aeruginosa*	PDC (AmpC) mutations/overexpression	In vitro Treatment-emergent	Treatment-emergent resistance despite low MPC; potential treatment failure
CFP-ETZ	*K. pneumoniae* and other Enterobacterales	MBLs; KPC variants; OmpK35/OmpK36 alterations/loss; AcrAB–TolC; PBP3 modifications; β-lactamase co-production	Baseline In vitro	Porin status may outweigh β-lactamase profile; AST before use in CPE
CFD	Enterobacterales *P. aeruginosa* *A. baumannii*	KPC, AmpC, SHV/PER, NDM variants; OXA-427; siderophore-receptor mutations; porins; efflux; PBP3; heteroresistance	Baseline In vitro	Heteroresistance missed by standard AST; repeat AST during therapy
PLZ	Enterobacterales, particularly NDM producers	16S rRNA methyltransferases (with *bla*NDM); rare AMEs	Baseline	Unreliable vs. NDM producers
ERV	*K. pneumoniae* *A. baumannii*	AcrAB–TolC; AdeABC; *tet*(X)	Baseline	Treatment-emergent resistance insufficiently characterized

The information presented in this table was obtained from the sources cited in the main text within the respective sections addressing each therapeutic option. Abbreviations: CAZ-AVI–ceftazidime/avibactam; ATM-AVI–aztreonam/avibactam; IMI-REL–imipenem-cilastatin/relebactam; SUL-DUR–sulbactam–durlobactam; CFP-ZID–cefepime/zidebactam; MEM-VAB–meropenem/vaborbactam; CTL-TZB–ceftolozane/tazobactam; CFP-ETZ–cefepime/enmetazobactam; CFD–cefiderocol; PLZ–plazomicin; ERV–eravacycline; KPC–*Klebsiella pneumoniae* carbapenemase; NDM–New Delhi metallo-β-lactamase; MBL–metallo-β-lactamase; VIM–Verona integron-encoded metallo-β-lactamase; IMP–imipenemase; OXA–oxacillinase; ESBL–extended-spectrum β-lactamase; SHV–sulfhydryl variable β-lactamase; CTX-M–cefotaximase-Munich; VEB–Vietnamese extended-spectrum β-lactamase; GES–Guiana extended-spectrum β-lactamase; PER–*Pseudomonas* extended resistance; SME–*Serratia marcescens* enzyme; CMY–cephamycinase; AmpC–AmpC β-lactamase; PDC–*Pseudomonas*-derived cephalosporinase; PBP–penicillin-binding protein; AMEs–aminoglycoside-modifying enzymes; CR-NME–carbapenem-resistant non-Morganellaceae Enterobacterales; ABC–*Acinetobacter baumannii–calcoaceticus* complex; MPC–mutant prevention concentration; AST–antimicrobial susceptibility testing; CPE–carbapenemase-producing Enterobacterales.

**Table 3:** Phenotypic assays for CP-CRO identification [62,81,207,208,209,210,211].

Assay	Variants	Method	Advantages	Limitations
MHT *		Growth-based	• easy to perform • low cost • good sensitivity for some carbapenemases (e.g., KPC)	• only for Enterobacterales • subjectivity in result interpretation • poor sensitivity for MBLs (e.g., NDM) and some OXAs • low specificity (false positives if ESBL/AmpC + permeability defects) • long TAT (18–24 h) • does not differentiate between carbapenemases or Ambler classes • can not detect multiple carbapenemases • no longer recommended by CLSI or EUCAST
**CDT**		Growth-based	• easy to perform • when a single carbapenemase is produced, easy to interpret • relatively low cost • differentiate between KPC, MBL and OXA-48	• only for Enterobacterales • variable sensitivity • difficult to interpret and poor sensitivity in multiple carbapenemase producers • long TAT (18–24 h)
**CIM**	CIM	Growth-based	• good sensitivity and specificity in carbapenemase-producing Enterobacterales • easy to perform and interpret • low cost • recommended by EUCAST	• only for Enterobacterales • concerns regarding OXAs, MBLs and poorly expressed carbapenemases • does not differentiate between carbapenemases or Ambler classes • cannot detect multiple carbapenemases • long TAT (18–24 h)
	mCIM	Growth-based	• improved sensitivity and specificity for all Ambler class carbapenemases in Enterobacterales • can be used to identify carbapenemase-producing PSAE • easy to perform and interpret • less subjective interpretation compared to other growth- or hydrolysis-based assays • low cost • recommended by CLSI	• does not differentiate between carbapenemases or Ambler classes • cannot detect multiple carbapenemases • long TAT (18–24 h)
	eCIM	Growth-based	• differentiation between SBLs and MBLs (when performed in parallel with mCIM) • easy to perform and interpret • low cost • recommended by CLSI	• only for Enterobacterales • false negative results if SBLs and MBLs are co-produced • long TAT (18–24 h)
	CIMTris	Growth-based	• improved sensitivity and specificity for detecting CP-PSAE and CP-AB	• long TAT (18–24 h)
**CarbaNP**	manual	Hydrolysis-based	• good sensitivity and specificity in detecting most carbapenemases in Enterobacterales and PSAE • good sensitivity and specificity in detecting plasmid-encoded OXA carbapenemases in AB (CarbAcineto NP test variant) • short TAT (30 min–2 h) • easy to perform • low cost • recommended by CLSI and EUCAST	• low sensitivity in detecting poorly carbapenem-hydrolysing carbapenemases (OXAs, GES, SME) and in mucoid strains • subjective interpretation in minor colour changes • pH meter needed • waste of materials, as requires preparation of short shelf-life reagents • does not differentiate between carbapenemases or Ambler classes (exception–Carba NP test II variant, which distinguishes KPC having added tazobactam and MBLs with EDTA)
	commercial	Hydrolysis-based	• good sensitivity in detecting most carbapenemases in Enterobacterales and PSAE • reagents in kits (no need for preparation, longer shelf life) • easy to perform • no need for additional equipment (e.g., pH meter) • short TAT (30 min–2 h)	• low sensitivity in detecting poorly carbapenem-hydrolysing carbapenemases (OXAs, GES, SME) and in mucoid strains • subjective interpretation in minor colour changes • higher costs (compared to manual version)
**MALDI-TOF MS**		Hydrolysis-based	• good sensitivity and specificity in detecting most carbapenemases in Enterobacterales, PSAE and AB (modified approach, adding bicarbonate) • can differentiate between Ambler class A (KPC), class B (MBLs) and class D (OXA-48) if class-specific inhibitors and temocillin are used • low cost, if the MALDI-TOF MS equipment is in place • short TAT (30 min–4 h)	• high costs for the equipment • different settings needed (from ones traditionally used for microbial species identification) • difficult interpretation of results, requiring expertise
**LFIA**		Antibody-based	• good sensitivity and specificity in detecting and differentiating between the most frequent carbapenemases in Enterobacterales (KPC, OXA-48, NDM, IMP, VIM) • can identify multiple carbapenemase producers • easy to perform • no additional equipment or expertise required • short TAT (≤15 min) • can be used directly on patient samples (e.g., positive blood cultures)	• higher costs (compared to other phenotypic assays) • poor discrimination between OXA-48-like enzymes • rare or unusual carbapenemases may be missed

Abbreviations: MHT–modified Hodge test; CDT–combined disc test; CIM–carbapenem inactivation method; mCIM–modified carbapenem inactivation method; eCIM–modified carbapenem inactivation method with EDTA; MALDI-TOF MS–Matrix-Assisted Laser Desorption/Ionization Time-of-Flight Mass Spectrometry; LFIA–lateral flow immunoassay; TAT–turnaround time; CLSI–Clinical and Laboratory Standards Institute; EUCAST–European Committee on Antimicrobial Susceptibility Testing; PSAE–*Pseudomonas aeruginosa*; AB–*Acinetobacter baumannii*; ESBL–extended spectrum betalactamase; KPC–*Klebsiella pneumoniae* carbapenemase; SBL–serine-betalactamase; MBL–metallo-betalactamase; OXA–oxacillinase; GES–Guiana extended-spectrum betalactamase; SME–*Serratia marcescens* enzyme; CIMTris–Tris-HCl–based carbapenem inactivation method; CarbAcineto NP–Carba NP test variant for *Acinetobacter* spp.; CP-CRO–carbapenemase-producing carbapenem-resistant organism(s); *, included for historical reference only.

## Data Availability

**No new data were created or analyzed in this study.**

## Abbreviations

The following abbreviations are used in this manuscript:

30S	30S ribosomal subunit
50S	50S ribosomal subunit
AB	*Acinetobacter baumannii*
ABC	*Acinetobacter baumannii–calcoaceticus* complex
ADC	Acinetobacter-derived cephalosporinase
AMEs	aminoglycoside-modifying enzymes
AMR	antimicrobial resistance
AMS	antimicrobial stewardship
AmpC	AmpC β-lactamase
ARI-1	Acinetobacter resistant to imipenem
AST	antimicrobial susceptibility testing
ATM–AVI	aztreonam–avibactam
BL/BLI	β-lactam/β-lactamase inhibitor
BLE	β-lactam enhancer
BSIs	bloodstream infections
CarbAcineto NP	Carba NP test variant for *Acinetobacter* spp.
carbaNP/Carba NP	Carba NP test (colorimetric hydrolysis-based assay)
CAZ–AVI	ceftazidime–avibactam
CDT	combined disc test
CFP–ETZ	cefepime–enmetazobactam
CFP-ZID	cefepime-zidebactam
CHDLs	carbapenem-hydrolysing class D β-lactamases
cIAI/cIAIs	complicated intra-abdominal infection(s)
CIM	carbapenem inactivation method
CIMTris	Tris-HCl-based carbapenem inactivation method
CLSI	Clinical and Laboratory Standards Institute
CMY	cephamycinase
COVID-19	coronavirus disease 2019
CP-CRO	carbapenemase-producing carbapenem-resistant organism(s)
non-CP CRO	non-carbapenemase-producing carbapenem-resistant organism(s)
CP-AB	carbapenemase-producing *Acinetobacter baumannii*
CP-PSAE	carbapenemase-producing *Pseudomonas aeruginosa*
CPE	carbapenemase-producing Enterobacterales
CR	carbapenem-resistant/carbapenem resistance
CR-GNB	carbapenem-resistant Gram-negative bacilli
CR-NME	carbapenem-resistant non-Morganellaceae Enterobacterales
CRAB	carbapenem-resistant *Acinetobacter baumannii*
CRE	carbapenem-resistant Enterobacterales
CRO/CROs	carbapenem-resistant organism(s)
CRPA	carbapenem-resistant *Pseudomonas aeruginosa*
CTL–TZB	ceftolozane–tazobactam
CTX-M	cefotaximase-Munich
cUTI/cUTIs	complicated urinary tract infection(s)
DBO/DBOs	diazabicyclooctane(s)
DNA	deoxyribonucleic acid
DTR	difficult-to-treat resistance
eCIM	EDTA-modified carbapenem inactivation method
EDTA	ethylenediaminetetraacetic acid
EMA	European Medicines Agency
ESBL/ESBLs	extended-spectrum β-lactamase(s)
EUCAST	European Committee on Antimicrobial Susceptibility Testing
FDA	U.S. Food and Drug Administration
GES	Guiana extended-spectrum β-lactamase
GIM	German imipenemase
GN	Gram-negative
GNB	Gram-negative bacilli
GP	Gram-positive
HAIs	healthcare-associated infections
HAP	hospital-acquired pneumonia
IMI	imipenem-hydrolyzing β-lactamase
IMI–REL	imipenem–cilastatin–relebactam
IMP	imipenemase
IPC	infection prevention and control
KPC	*Klebsiella pneumoniae* carbapenemase
LFIA	lateral flow immunoassay
MALDI-TOF MS	matrix-assisted laser desorption/ionization time-of-flight mass spectrometry
MBL/MBLs	metallo-β-lactamase(s)
mCIM	modified carbapenem inactivation method
MDR	multidrug-resistant/multidrug resistance
MDR-GNB	multidrug-resistant Gram-negative bacilli
MEM–VAB	meropenem–vaborbactam
MGEs	mobile genetic elements
MHT	modified Hodge test
MIC/MICs	minimum inhibitory concentration(s)
MPCs MTZ	mutant prevention concentrations metronidazole
N/A	not applicable
NDM	New Delhi metallo-β-lactamase
NF-GNB	non-fermenting Gram-negative bacilli
NGS	next-generation sequencing
NMC-A	non-metallo class A carbapenemase
NME	non-Morganellaceae Enterobacterales
OMPs	outer membrane proteins
OMVs	outer membrane vesicles
OXA/OXAs	oxacillinase(s)
PBP/PBPs	penicillin-binding protein(s)
PCR	polymerase chain reaction
PDC/PDCs	Pseudomonas-derived cephalosporinase(s)
PDR	pandrug-resistant/pandrug resistance
PER	Pseudomonas extended resistance
PK/PD	pharmacokinetic/pharmacodynamic
PSAE	*Pseudomonas aeruginosa*
RND	resistance-nodulation-division
rRNA	ribosomal RNA
SBL/SBLs	serine β-lactamase(s)
SFC	*Serratia fonticola* carbapenemase
SHV	sulfhydryl variable β-lactamase
SIM	Seoul imipenemase
SME	*Serratia marcescens* enzyme
SPM	São Paulo metallo-β-lactamase
SSTIs	skin and soft tissue infections
ST	sequence type
SUL–DUR	sulbactam–durlobactam
TAT	turnaround time
TEM	Temoneira β-lactamase
UTI/UTIs	urinary tract infection(s)
VAB	vaborbactam
VAP	ventilator-associated pneumonia
VEB	Vietnamese extended-spectrum β-lactamase
VIM	Verona integron-encoded metallo-β-lactamase
WGS	whole-genome sequencing
WHO	World Health Organization
XDR	extensively drug-resistant/extensive drug resistance

## Nomenclature

Gene, enzyme, porin and efflux-pump designations also used: AcrAB–TolC, AdeABC, adeJ; *bla*IMP, *bla*KPC, *bla*NDM, *bla*OXA, *bla*VIM; CphA, GOB-1, L1; MexAB–OprM, MexCD–OprJ, MexXY; OmpC, OmpF, OmpK35, OmpK36, OprD; PBP1b, PBP1c, PBP3; tet(X); 16S rRNA; Zn^2+^.
